# The N-terminal extension of ribosomal protein Rps2 shapes its early fate through Chaperone–Importin competition and arginine methylation

**DOI:** 10.1016/j.jbc.2026.113481

**Published:** 2026-08-25

**Authors:** Maximilian Mack, Benjamin Pillet, Nicole Holton, Ingrid Zierler, Stephanie Himpich, Phyllis Jessen, Markus C. Wahl, Dieter Kressler, Brigitte Pertschy

**Affiliations:** 1 Institute of Molecular Biosciences, University of Graz, Graz, Austria; 2 BioTechMed-Graz, Graz, Austria; 3 Department of Biology, University of Fribourg, Fribourg, Switzerland; 4 Laboratory of Structural Biochemistry, Institute of Chemistry and Biochemistry, Freie Universität Berlin, Berlin, Germany; 5 Macromolecular Crystallography, Helmholtz-Zentrum Berlin für Materialien und Energie, Berlin, Germany

**Keywords:** *Saccharomyces cerevisiae*, ribosome assembly, post-translational modification (PTM), protein–protein interaction, nuclear transport, protein methylation, protein arginine methylation, rps2, tsr4, hmt1

## Abstract

Efficient nuclear import of ribosomal proteins is essential for the timely assembly of ribosomal subunit precursors in the nucleus and progression of ribosome biogenesis. Several ribosomal proteins are escorted to the nucleus by dedicated chaperones, which shield their interaction surfaces and assist their delivery. Here, we characterize the N-terminal extension of the small subunit ribosomal protein Rps2 as a regulatory hub that integrates binding of its dedicated chaperone Tsr4, recognition by its importin Pse1, and arginine methylation. By mapping Pse1’s interaction interface on Rps2’s N-terminal extension, we identified arginine 11 (R11), a known methylation site, as a critical residue. We demonstrate that Rps2 is already methylated while associated with Tsr4, indicating that methylation occurs at an early stage of the Rps2 assembly path prior to nuclear import. We further show that the chaperone Tsr4 and the importin Pse1 compete for binding to the Rps2 N-terminal extension. While Tsr4 binds this region with higher affinity than Pse1, Pse1 also contacts additional regions within full-length Rps2. Arginine methylation of Rps2 modestly reduces Pse1 binding to the Rps2 N-terminal extension, while having little effect on Tsr4 binding, suggesting that this modification can influence importin recognition without disrupting chaperone association. Finally, we find that Rps2 methylation increases at low temperature and that loss of Hmt1-mediated methylation exacerbates translational fidelity defects in an *rps2* mutant background. Together, our findings reveal how a ribosomal protein N-terminal extension coordinates sequential interactions and post-translational modification events that shape the early fate of Rps2 and support accurate ribosome function.

Eukaryotic ribosome assembly is a highly coordinated process that spans several compartments of the cell. Efficient ribosome synthesis depends on the timely expression and precise subcellular localization of all participating components. In the model eukaryote *Saccharomyces cerevisiae* (yeast), ribosome assembly involves four ribosomal RNAs (rRNAs), 79 ribosomal proteins (r-proteins), and over 200 ribosome assembly factors, collectively ensuring the production of approximately 2000 to 4000 ribosomes per minute (1, 2, 3). A large number of ribosome assembly factors and nearly all newly synthesized r-proteins must be imported from the cytosol into the nucleus to participate in assembly.

Most r-proteins contain clusters of positively charged amino acids and unstructured extensions, which make the r-proteins prone to aggregation as long as they are in their free form and not incorporated into ribosomes, requiring mechanisms to protect them on the path until incorporation into ribosomal particles (4). Several r-proteins have their dedicated chaperones, which protect the highly basic and aggregation-prone r-proteins from non-productive interactions. Many of these dedicated chaperones bind r-proteins already co-translationally, while they are being synthesized (5, 6, 7, 8, 9, 10, 11, 12, 13, 14).

Although most r-proteins are smaller than 40 kDa and theoretically capable of passive diffusion through the nuclear pore complex (NPC), specific import receptors, known as importins, mediate their active translocation, likely increasing the efficiency of nuclear import (15). Importins recognize their cargo proteins *via* nuclear localization signals (NLSs), with different importins preferring different NLS motifs. Nevertheless, many NLSs are recognized by multiple importins, which can act redundantly, for example in r-protein or histone import (4, 16, 17, 18, 19, 20). Proteins frequently harbor multiple NLSs, potentially to enhance import efficiency and/or as a backup mechanism (7, 21, 22, 23). Although nucleocytoplasmic transport is fundamental to many cellular processes, comparatively little is known about how nuclear import is regulated. Importin-binding affinity generally correlates with nuclear localization efficiency (24), and several studies have demonstrated that post-translational modifications, such as phosphorylation near or within NLSs, can modulate importin binding (25, 26, 27, 28, 29).

One post-translational modification whose role in nuclear import is less well understood is arginine methylation. Protein arginine methyltransferases (PRMTs) transfer methyl groups from S-adenosylmethionine to arginine residues, generating monomethylarginine (MMA), symmetric dimethylarginine (SDMA), or asymmetric dimethylarginine (ADMA) (30, 31, 32). In yeast, Hmt1 is the predominant PRMT and catalyzes sequential mono- and asymmetric dimethylation of arginine residues (33, 34). Approximately 0.25% of all arginine residues in yeast proteins are methylated, with ADMA accounting for ∼85% and MMA for ∼15% of these modifications (35). Arginine methylation frequently occurs in structurally disordered repetitive regions rich in arginine (R) and glycine (G) residues, such as RG, RGG, RXG, and RXR sequences (30, 31, 36). However, methylation motifs are still not fully explored, as many reported arginine methylation sites do not match any so far reported consensus (32).

We recently characterized the early stages of the life cycle of the yeast r-protein Rps2 (also termed uS5 (37)). Rps2 contains a eukaryote-specific, positively charged, RG-rich N-terminal extension that serves as a multifunctional interaction platform (Fig. S1). During translation, this extension is recognized co-translationally by the dedicated chaperone Tsr4, which shields the highly basic N-terminal region (10, 38). Although the timing of Tsr4 dissociation remains unclear, direct evidence for nuclear localization of Tsr4 is lacking, suggesting that it is released before Rps2 enters the nucleus. We found that nuclear import of Rps2 is mediated by the importin Pse1/Kap121 (37). Pse1 recognizes two independent NLSs within Rps2: one located between residues 76 and 145, in which residues R95, R97, and K99 are critical for importin binding, and a second NLS within the N-terminal extension that overlaps with the Tsr4-binding site (37). In addition to binding the dedicated chaperone Tsr4 and the importin Pse1, the N-terminal extension of Rps2 is also recognized by the arginine methyltransferase Hmt1, which methylates one or two arginine residues in this region. Both MMA and ADMA have been detected on Rps2, although their relative abundance has not been determined (39, 40, 41). The functional significance of these arginine methylation events remains unknown.

Together, these observations support the following model for the early life cycle of Rps2. Newly synthesized Rps2 is first captured co-translationally by Tsr4 in the cytoplasm (10, 38). Following dissociation of Tsr4, Rps2 is imported into the nucleus by Pse1, which recognizes both the central and N-terminal NLSs (37). As with other importin cargoes, binding of RanGTP to Pse1 is expected to trigger cargo release after nuclear entry (16). Rps2 is subsequently incorporated into late pre-40S ribosomal particles, where it contributes to formation of the central pseudoknot, a highly conserved RNA junction that connects multiple rRNA domains and forms the mRNA entry channel (42, 43, 44). Consistent with positioning of Rps2 at this functionally important site, it plays a critical role in maintaining translational accuracy (45, 46).

At an as yet unknown stage of this pathway, the N-terminal extension is methylated by Hmt1 (39, 40, 41). Here, we investigated the interplay between arginine methylation, nuclear import, and chaperone interaction during the early fate of Rps2. We identify arginine 11 (R11), a known Hmt1 methylation site, as a determinant of Pse1-mediated nuclear import and show that Tsr4 and Pse1 compete for binding to the Rps2 N-terminal extension. Furthermore, we demonstrate that Rps2 methylation occurs at an early stage of its maturation, while associated with Tsr4, and weakens Pse1 binding without disrupting the interaction with Tsr4. Finally, we provide evidence that Hmt1-mediated methylation contributes to translational fidelity when Rps2 function is compromised.

## Results

### Rps2 residues R11 and R17 are crucial for the function of its N-terminal NLS

We previously demonstrated that Rps2 contains both an internal and an N-terminal NLS, with each being recognized by the importin Pse1 (37). Mutation of eight positively charged residues within the N-terminal region (residues 10–28) abolishes its NLS function (37). However, the specific contribution of individual residues within the N-terminal NLS region remained uncharacterized. To determine which of the eight basic residues in the N-terminal NLS region are critical for Pse1 binding, we employed AlphaFold 3 to predict the structure of the Rps2–Pse1 complex (Figs. 1*A* and S2) (47). In the top five predicted models, the N-terminal ∼28 residues of Rps2 appeared intrinsically disordered and displayed lower pLDDT scores than the remainder of the protein (Fig. S3). While the positioning of this N-terminal Rps2 extension varied among the models, the two highest-ranked predictions suggested a similar mode of interaction with Pse1. In these models, the interface also involves salt bridges formed by two of the eight candidate basic residues, R11 and R17, of Rps2 with the negatively charged residues D353, E396, and E480, which constitute key residues of the known Pse1 NLS interaction surface (48) (Figs. 1*A* and S2).

**Figure 1:**
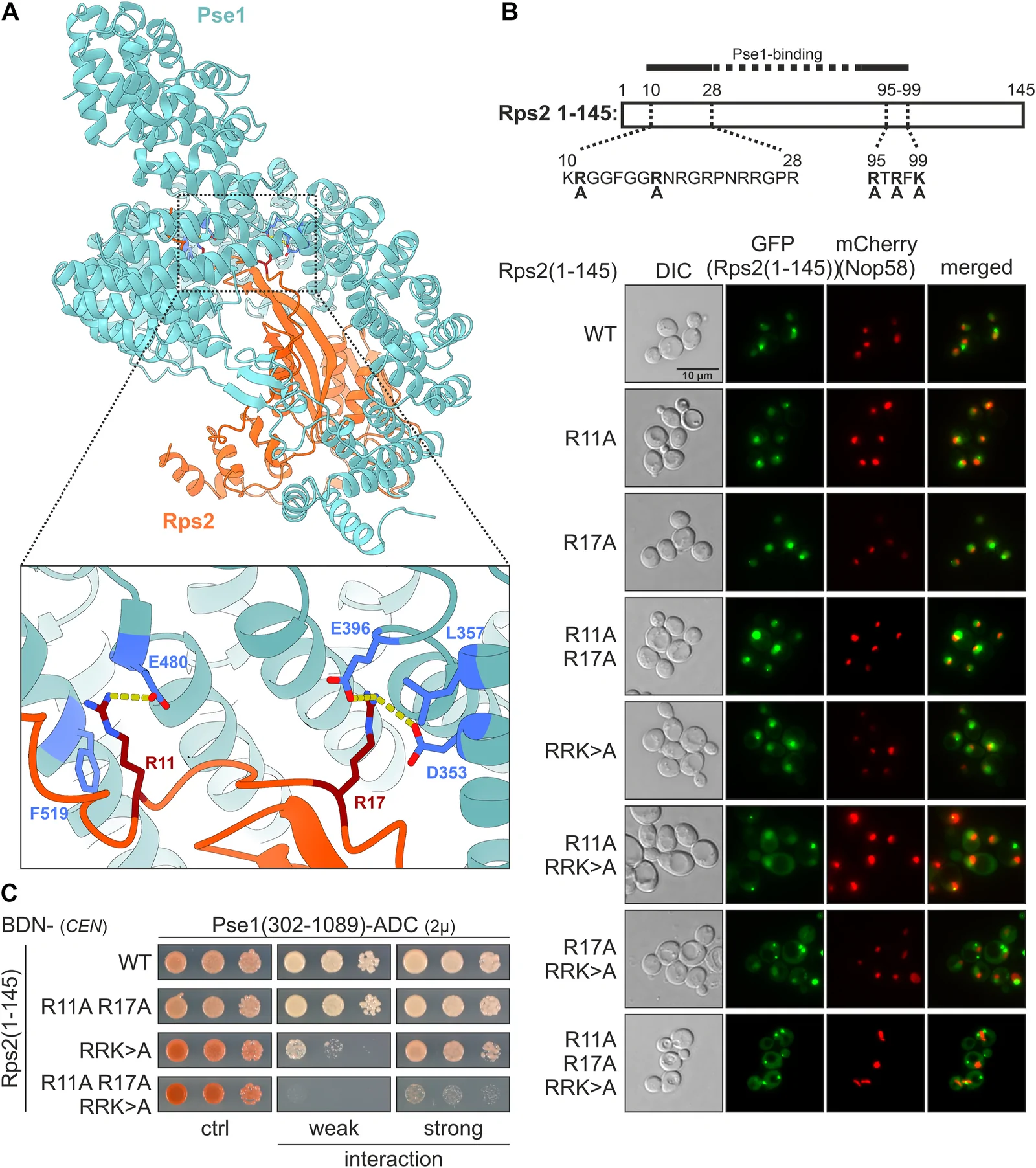
**Rps2 R11 and R17 are key residues for Pse1 interaction.***A*, *top* AlphaFold 3 prediction model (model 1) of the complex formed between Pse1 and Rps2: Pse1 is represented in teal, while Rps2 is shown in orange. In the zoom-in view, Pse1 residues L357, D363, E396, E480, and F519, which have been previously identified as critical for cargo recognition, are marked in blue, and Rps2 R11 and R17 are marked in dark red. Distances between these residues of ∼3 Ångström or less are regarded as potential interactions and are indicated by *dashed lines*. *B*, fluorescence microscopy of strains expressing Nop58-yEmCherry (nucleolar marker) as well as C-terminal 3xyEGFP fusions of Rps2(1–145) or the indicated Rps2(1–145) variants. Tested amino acid exchanges are also indicated in the scheme of the Rps2(1–145) fragment (*upper panel*). DIC, differential interference contrast. *C*, yeast two-hybrid (Y2H) assays between Pse1(302–1089), C-terminally fused to the Gal4 activation domain (ADC), and Rps2(1–145) and indicated mutant variants thereof, N-terminally fused to the Gal4 DNA-binding domain (BDN). The letters C and N indicate the position of the Gal4 domain relative to the protein of interest. Growth on SDC-Leu-Trp served as control (ctrl). Weak Y2H interactions (*HIS3* reporter) were detected by growth on SDC-His-Leu-Trp, strong interactions (*ADE2* reporter) by growth on SDC-Ade-Leu-Trp plates.

To experimentally validate the predicted contribution of Rps2 R11 and R17 to the interaction with Pse1, we employed our previously established Rps2(1–145)-3xyEGFP reporter, which contains both NLS elements and localizes to the nucleus (37). We assessed the subcellular localization of this reporter following individual or combined mutation of R11 and R17 to alanine, both alone and in combination with a disrupted internal NLS (R95A, R97A, and K99A; referred to as RRK>A).

As expected, single or double alanine substitutions of R11 and R17 did not affect nuclear localization when the internal NLS remained intact, consistent with sufficient import driven by the internal NLS (Fig. 1*B*). Similarly, and in agreement with our previous findings (37), mutating the internal NLS alone (RRK>A) led to nuclear localization (Fig. 1*B*), presumably mediated by the intact N-terminal NLS. When either R11A or R17A were combined with mutation of the internal NLS, a partial mislocalization of the Rps2(1–145)-3xyEGFP reporter to the cytoplasm could already be observed. The R11A/R17A double mutation in combination with the RRK>A internal NLS mutation resulted in an even more pronounced shift of the signal to the cytoplasm (Fig. 1*B*). These results strongly support that both R11 and R17 are essential for the function of the N-terminal NLS *in vivo*. As noted previously (37), we frequently observed small dot-like GFP signals, particularly for reporter fusion proteins exhibiting cytoplasmic mislocalization. These likely represent aggregates formed due to reduced solubility of the mutant protein, potentially as a consequence of impaired interaction with Pse1.

To directly test whether the observed mislocalization correlates with reduced importin binding, we performed yeast two-hybrid (Y2H) assays using a previously described Pse1 truncation lacking the 301 N-terminal residues (Pse1(302–1089); previously termed Pse1.302C) (37). This truncation lacks nearly all RanGTP-interacting residues (48, 49), thereby preventing efficient RanGTP-mediated cargo release in the nucleus and enabling stable detection of the Pse1-Rps2 interaction by Y2H (37). In the Y2H assay, plasmids expressing the two proteins of interest, each fused to complementary domains of the Gal4 transcription factor, are introduced into a reporter strain. Interaction between the two proteins reconstitutes a functional Gal4 transcription factor, thereby activating expression of reporter genes that can be monitored by growth on selective media. Growth on -His plates indicates detectable protein interaction (referred to as weak interaction in Fig. 1*C*), whereas growth on the more stringent -Ade plates is only observed for stronger interactions. In addition, differences in interaction strength can be inferred from colony size, as stronger interactions generally result in faster growth on the reporter plates. In agreement with the localization data, both wild-type Rps2(1–145) and the R11A/R17A variant showed a strong interaction with Pse1(302–1089). While the interaction was weakened in the Rps2(1–145) RRK>A variant, the R11A/R17A double mutant variant that additionally carried the RRK>A internal NLS mutation almost completely lost interaction with Pse1(302–1089) (Fig. 1*C*). In conclusion, our data demonstrate that Rps2 residues R11 and R17 constitute critical residues of the N-terminal NLS *in vivo*.

### Rps2 is arginine-methylated by Hmt1 early in its lifetime

Intriguingly, one of the two key residues of the Rps2 N-terminal NLS, R11, is a known target of monomethylation and asymmetric dimethylation by the arginine methyltransferase Hmt1 (39, 40, 41). In addition, R17 has been suggested as a second methylation site in a previous study (41). These observations raised the hypothesis that Rps2 arginine methylation might influence its interaction with Pse1.

However, for methylation to affect Pse1 binding, Rps2 would need to be methylated before or during the interaction with importin. We therefore investigated the complexes in which methylated Rps2 is present. During its ribosome assembly path, Rps2 progresses through a defined series of intermediates: it is initially bound co-translationally by Tsr4, subsequently interacts with the importin Pse1 for nuclear import, and is finally incorporated into pre-40S particles that maturate into functional 40S ribosomal subunits. Thus, identifying intermediates containing methylated Rps2 allows us to infer at which stage of the Rps2 ribosome assembly pathway Hmt1-dependent methylation occurs.

As a first step, we sought to establish a western blot-based assay for the detection of Rps2 R11 methylation. To evaluate the specificity and sensitivity of commercially available antibodies, we used synthetic peptides corresponding to Rps2 residues 5 to 28, followed by a lysine conjugated to a Cy5 fluorophore. Peptides with R11 in unmethylated, monomethylated (labeled MMA), and asymmetrically dimethylated (labeled ADMA) forms were tested. The anti-MMA antibody (no. 8711 Cell Signaling Technology) specifically recognized the monomethylated peptide, yielding signals down to 0.08 nmoles of peptide (Fig. 2*A*), while no signal was detected for the unmethylated version. The ADMA-modified peptide was also detected, albeit with much lower sensitivity, with a weak signal only at 10 nmoles (Fig. 2*A*). In contrast, one tested MMA/DMA-specific antibody and two tested ADMA-specific antibodies failed to detect the ADMA-modified peptide (Fig. S4). Based on these results, we proceeded with the anti-MMA antibody, while noting its preference for detecting monomethylated R11 over the asymmetrically dimethylated form.

**Figure 2:**
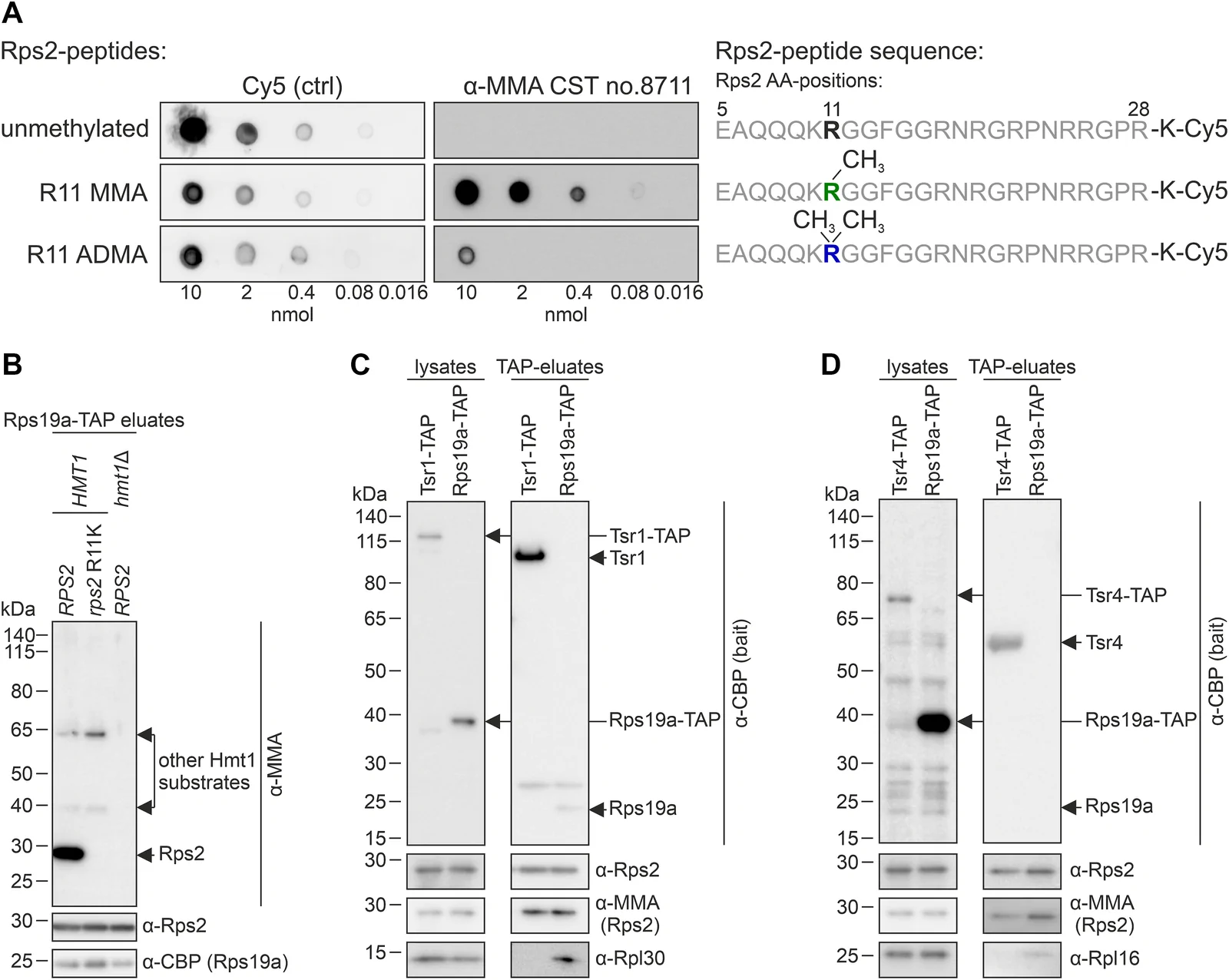
**Rps2 R11 is arginine-methylated by Hmt1 early in its lifetime.***A*, Cy5-labeled peptides corresponding to Rps2(5–28) with different R11 methylation states (unmethylated; MMA, monomethylarginine; ADMA, asymmetric dimethylarginine) were spotted onto a PVDF membrane at the indicated amounts. *Left panels*: Cy5 fluorescence was detected as control for peptide loading. *Right panels*: Methylation was detected using an anti-MMA antibody. Note that the identical panel is shown in Figure S4 to facilitate comparison with blots obtained using other antibodies. *B*, Western blot analysis of Rps19a-TAP eluates purified from wild-type (*RPS2*), *rps2* R11K, and *hmt1*Δ cells using the indicated antibodies. In addition to Rps2, two faint bands were detected in *RPS2* and *rps2* R11K samples that were absent in *hmt1*Δ samples, suggesting they represent additional Hmt1 methylation substrates associated with ribosomes. *C and D*, Western blot analysis of lysates and eluates from Tsr1-TAP compared to Rps19a-TAP purification (*C*) and from Tsr4-TAP compared to Rps19a-TAP purification (*D*). Protein loading was adjusted to achieve approximately equal amounts of Rps2 per lane. Rpl30 (*C*) and Rpl16 (*D*) were used as controls to verify that the Rps2 signal in the Tsr1-TAP or Tsr4-TAP eluates does not originate from contamination with mature ribosomes. These large subunit r-proteins were only detected in mature ribosomes present in the Rps19a-TAP eluates.

We next used Western blotting to assess R11 methylation of Rps2 in mature ribosomes purified *via* Rps19-TAP. A strong anti-MMA signal was observed that co-migrated with the band detected by an Rps2-specific antibody, indicating that Rps2 is methylated when incorporated into ribosomes (Fig. 2*B*). This signal was abolished in ribosomes purified from an *hmt1*Δ strain and from a strain expressing the R11K variant of Rps2, confirming that the observed anti-MMA signal specifically reflects R11 methylation by Hmt1 (Fig. 2*B*).

To determine whether Rps2 is methylated prior to its presence in mature ribosomes, we purified Rps2 from distinct stages of its life cycle. Specifically, we isolated the early Rps2-Tsr4 chaperone complex using Tsr4-TAP and the pool of Rps2 associated with pre-40S particles using the ribosome assembly factor Tsr1-TAP as bait. Remarkably, Rps2 was methylated not only in pre-40S particles (Fig. 2*C*) but also in the Tsr4-bound complex (Fig. 2*D*). As Tsr4 associates with Rps2 co-translationally and dissociates before Rps2 incorporation into pre-ribosomal particles, these findings indicate that Rps2 R11 methylation is already present at an early stage in the lifetime of Rps2.

### Arginine 11 methylation reduces Rps2(5–28) binding to importin Pse1 while not affecting Tsr4 interaction

As Rps2 is already methylated early in its lifetime while it is bound to its dedicated chaperone Tsr4 (Fig. 2*D*), we next asked whether modification of R11 modulates the interaction of Rps2 with Tsr4. Synthetic Cy5-labeled Rps2(5–28) peptides, which were either unmethylated or carried MMA or ADMA at position 11 (Fig. 3*A*), were titrated with serial dilutions of recombinant Tsr4 purified from *Escherichia coli* (Fig. S5*A*). We utilized the observation that binding to Tsr4 leads to an increase in the fluorescence intensity of the Cy5-labeled peptides at 670 nm to record binding curves (Fig. 3*B*). Global fits of three replicate-binding curves obtained with independent Tsr4 preparations yielded dissociation constants (*K*_*d*_) of 159.3 ± 41.1 nM (R11 unmodified), 135.4 ± 26.3 nM (R11 MMA), and 183.4 ± 48.9 nM (R11 ADMA), suggesting that R11 methylation does not significantly influence Tsr4 affinity for Rps2 (Fig. 3*B*).

**Figure 3:**
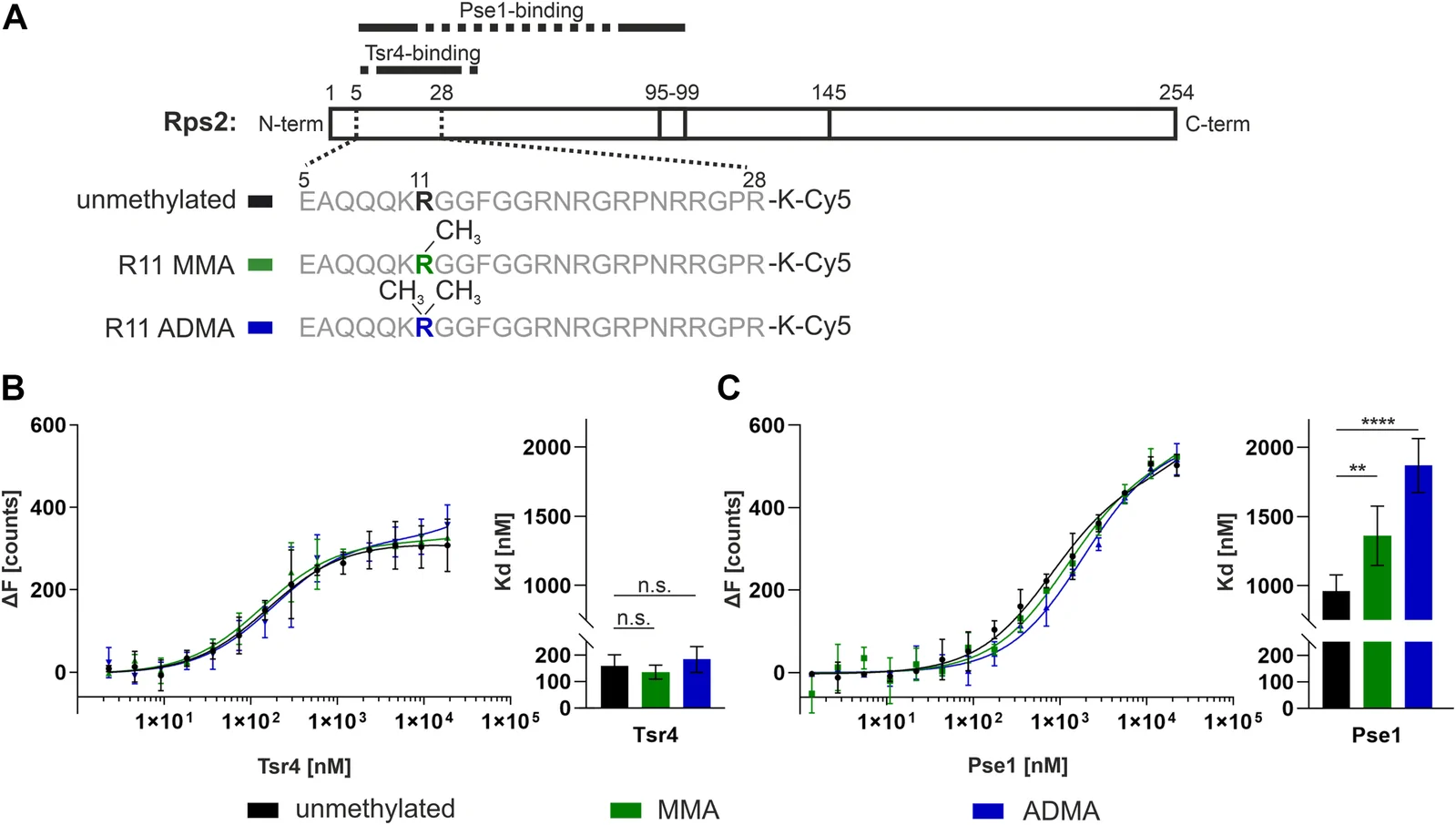
**R11 methylation reduces the affinity of Rps2(5–28) for binding to the importin Pse1.***A*, schematic representation of Rps2. Binding sites of Tsr4 and Pse1 are indicated with *bold lines*. The segment of the N-terminal region used for synthetic peptide production is indicated in *dashed lines* and its amino acid sequence and R11 methylation states are additionally indicated *below*. *B and C*, dose–response curves of Rps2 peptide interactions with either Tsr4 (*B*) or Pse1 (*C*). Binding events elicit a change of fluorescence of each Rps2 peptide variant upon addition of Tsr4 and Pse1 by 300 and 500 raw fluorescence counts, respectively. Mean values and standard deviations from three biological replicates are shown. *K*_*d*_ values are depicted in a bar chart next to each dose-response curve. An ANOVA, followed by Dunnett’s multiple comparison test, was performed to assess significance (∗∗ = *p*-value ≤ 0.01, ∗∗∗∗ = *p*-value ≤ 0.0001, n.s. = not significant).

We next investigated the effect of R11 methylation on the interaction with Rps2’s importin Pse1 by again following the fluorescence change of Cy5-labeled Rps2(5–28) peptides at 670 nm (Fig. 3*C*). Recombinant Pse1 (Fig. S5*B*) bound the unmethylated Rps2 peptide with a higher dissociation constant than Tsr4 (*K*_*d*_ of 960.6 ± 117.0 nM), suggesting weaker binding of the unstructured N-terminal extension of Rps2 by Pse1 than by Tsr4. Interestingly, methylation progressively reduced the interaction: the MMA peptide displayed a *K*_*d*_ of 1361.3 ± 214.2 nM, whereas the ADMA peptide showed a *K*_*d*_ of 1870.4 ± 194.5 nM (Fig. 3*C*). Together, our data reveal that both Tsr4 and Pse1 bind to the unstructured N-terminal extension of Rps2, with Tsr4 binding stronger. While the interaction of Rps2’s N-terminal extension with Tsr4 is largely unaffected by R11 methylation, methylation weakens its association with Pse1.

### Pse1 and Tsr4 compete for binding to the N-terminal extension of Rps2

Given that both Pse1 and Tsr4 individually interact with the N-terminal segment of Rps2, we next asked whether the two proteins can bind simultaneously to this region, or whether they compete for binding. To address this, we employed analytical size exclusion chromatography to examine the formation of binary or ternary complexes involving Rps2(5–28), Tsr4, and Pse1 (Fig. S6*A*). As expected from their molecular weights, Pse1 (121 kDa) eluted earlier than Tsr4 (46 kDa). In a mixed sample containing both proteins, two distinct elution peaks corresponding to the individual elution volumes of Tsr4 and Pse1 were observed, indicating that the two proteins do not form a stable complex in the absence of Rps2 (Fig. S6*A*, left panel). When Cy5-labeled Rps2(5–28) peptide was added separately to either Tsr4 or Pse1, the Cy5 fluorescent signal co-eluted with each respective protein peak, confirming that both proteins interact with the peptide (Fig. S6*A*, right panel). However, in samples containing all three partners, the Cy5 signal predominantly co-eluted with Tsr4, and no peak corresponding to a ternary complex was detected. This suggests that Tsr4 and Pse1 cannot simultaneously bind the Rps2 N-terminal extension and that Rps2(5–28) preferentially associates with Tsr4 under these conditions.

To directly test whether Tsr4 and Pse1 compete for Rps2 binding under equilibrium conditions, we next performed fluorescence polarization (FP) assays (Fig. 4*A*). We first determined the FP response of the Cy5-labeled unmodified and R11 ADMA-modified Rps2(5–28) peptides upon binding to each protein individually. Binding of both Pse1 and Tsr4 to the Cy5-labeled peptides resulted in an increase in FP relative to the unbound state. At saturating concentrations, Pse1 binding produced an FP increase approximately twice that observed for Tsr4 binding for both peptides.

**Figure 4:**
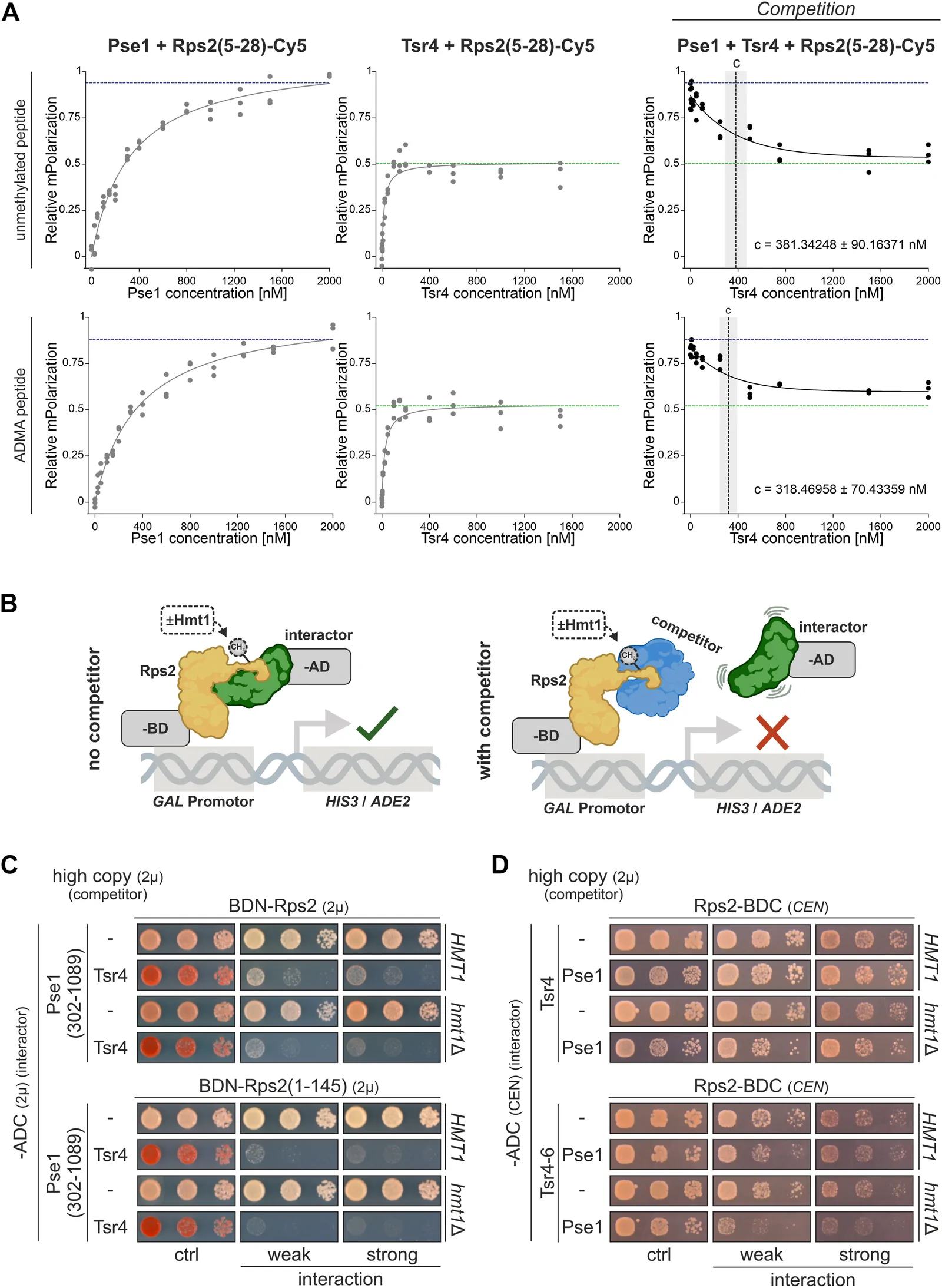
**Competition between Pse1 and Tsr4 for Rps2 binding.***A*, fluorescence polarization (FP)-based binding and competition assays. *Left* and *middle panels*, binding curves showing the interaction of Cy5-labeled unmodified (*upper panels*) or ADMA-modified (*lower panels*) Rps2(5–28) peptides with Pse1 and Tsr4. *Right panels*, competition experiment, in which the Cy5-labeled peptides were incubated with a saturating concentration of Pse1, followed by titration of increasing concentrations of Tsr4. FP values were normalized to the signal of the unbound peptide (0) and the mean FP value at the highest Pse1 concentration (1). The plateau values of Pse1 and Tsr4 are indicated by *blue* and *green lines*, respectively. *Dashed vertical lines* indicate the fitted decay constants. *B*, schematic representation of the Y2H competition assay used in (*C*) and (*D*). The assay monitors whether the interaction between Rps2 and its interaction partner, detected *via* reporter gene transcription (*HIS3/ADE2*), is disrupted by overexpression of a competitor that also binds to Rps2. Additionally, the effect of the presence or absence of Hmt1, and thus of the methylation status of R11, on the interaction between Rps2 and its interaction partner are assessed. *C*, Y2H assays assessing interactions between Pse1(302–1089) (C-terminally fused to the Gal4 activation domain, ADC) and either full-length Rps2 or Rps2(1–145) (both N-terminally fused to the Gal4 DNA-binding domain, BDN) in the absence (−) or presence of Tsr4 expressed from a high-copy plasmid in the wild-type or *hmt1*Δ Y2H reporter strain. *D*, Y2H assays assessing interactions between Tsr4 or the Tsr4-6 variant (C-terminally fused to the Gal4 activation domain, ADC) and full-length Rps2 (C-terminally fused to the Gal4 DNA-binding domain, BDC) in the absence (−) or presence of Pse1 expressed from a high-copy plasmid in the wild-type or *hmt1*Δ Y2H reporter strain. For panels (*C*) and (*D*), growth on SDC-His-Leu-Trp-Ura plates indicates a weak interaction, and growth on SDC-Ade-Leu-Trp-Ura plates indicates a strong interaction. SDC-Leu-Trp-Ura served as growth control.

To assess competitions, 5 nM Cy5-labeled unmodified or ADMA-modified Rps2(5–28) peptide were pre-incubated with a saturating concentration of Pse1 (2000 nM), followed by titration of increasing concentrations of Tsr4. As the Tsr4 concentration increased, FP gradually decreased from the Pse1-bound value toward the lower Tsr4-bound value, indicating displacement of Pse1 from the peptide in favor of Tsr4 binding. These results demonstrate that Tsr4 effectively competes with Pse1 for binding to the Rps2 N-terminal extension, consistent with its higher affinity for this region. The fitted decay constants were approximately 381 ±90 nM Tsr4 for the unmodified peptide and 318 ±70 nM for the ADMA-modified peptide in the representative experiment shown in Fig. 4*A*. Across three independent experiments, no consistent difference in the competition curves was observed between the unmodified and ADMA-modified peptides. Thus, these experiments do not provide evidence for a discernible effect of R11 methylation on Pse1–Tsr4 competition under the conditions tested.

While the FP assay demonstrated that Tsr4 can displace Pse1 from the isolated Rps2 N-terminal peptide under equilibrium conditions, it remained unclear whether this competition also occurs in the context of full-length Rps2 in living cells. Because Pse1 additionally recognizes an internal binding site within Rps2 (Fig. 1*B*) (37), competition *in vivo* cannot be inferred directly from the peptide-based assay. We therefore performed Y2H assays using Rps2 as bait and either Pse1(302–1089) or Tsr4 as prey, in the presence of the respective other binding partner as a competitor. Additionally, we tested whether deletion of *HMT1*, and thus loss of Rps2 R11 methylation, influences this competition (Fig. 4*B*). Overexpression of Tsr4 from a high-copy plasmid markedly reduced the interaction between Rps2 and Pse1(302–1089), both in the case of full-length Rps2 and of the Rps2(1–145) fragment, which exhibits a slightly stronger Y2H interaction. This competition also occurred in a *hmt1*Δ strain (Fig. 4*C*).

A limitation of the abovementioned assay is that detection of the Y2H interaction between Rps2 and Pse1 requires usage of the truncated Pse1(302–1089) variant. While this variant was generated with the goal to reduce RanGTP binding and thus stabilize cargo association, the truncation might also partially impair cargo binding. To overcome this limitation and evaluate interactions using Rps2, Tsr4, and Pse1 full-length proteins, we evaluated the reciprocal Y2H interaction, this time using Rps2 as bait, Tsr4 as prey, and full-length Pse1 as competitor (Fig. 4*D*). Increasing Pse1 levels by expression from a high-copy *PSE1* plasmid did not significantly affect the interaction between Rps2 and Tsr4, suggesting that full-length Pse1 does not effectively compete with Tsr4 under these conditions. A likely explanation is that, unlike the truncated construct, full-length Pse1 retains its ability to bind RanGTP and is therefore expected to dissociate rapidly from its cargo in the nucleus, where the Y2H reporter proteins are localized.

To sensitize the assay, we employed a Tsr4 variant (Tsr4-6), obtained by random PCR mutagenesis, which carries four amino acid substitutions (L97P, N126D, K146E, and T270A). Tsr4-6 is a temperature-sensitive mutant, exhibiting a severe growth defect (Fig. S6*B*) and reduced Tsr4 protein levels (Fig. S6*C*) at 37 °C, consistent with decreased protein stability under these conditions. However, at the temperature used for the Y2H assay (30 °C), Tsr4-6 supported normal growth (Fig. S6*B*). Y2H analysis revealed that Tsr4-6 binds Rps2 slightly weaker than wild-type Tsr4, as reflected by reduced colony size on both -His (weak interaction) and -Ade (strong interaction) selection plates (Fig. 4*D*). Under these conditions, overexpression of Pse1 still had only a small effect on the interaction between Tsr4-6 and Rps2. Notably, however, in an *hmt1*Δ background, Pse1 overexpression substantially weakened the Tsr4-6-Rps2 interaction, suggesting that the absence of Rps2 methylation enhances the ability of Pse1 to compete with Tsr4.

Thus, while the overall competition between Tsr4 and Pse1 is largely determined by their intrinsic binding properties, Hmt1-dependent methylation of Rps2 appears to subtly modulate the competitive balance in favor of Tsr4.

### Hmt1-dependent methylation contributes to translational fidelity of Rps2 variants at low temperatures

Our findings suggest a role of Hmt1-mediated methylation in the import of Rps2, potentially through modulating the competition between Pse1 and Tsr4 for Rps2 binding. Given that *HMT1* is a non-essential gene, cells must tolerate the absence of Rps2 arginine methylation under normal conditions. However, we hypothesized that a partial loss of Rps2 function might increase the requirement for Hmt1 function. To test this, we sought to identify *rps2* mutants that exhibit synergistic growth defects when combined with *hmt1*Δ (Fig. 5*A*). For this purpose, we generated a *hmt1*Δ *rps2*Δ double knockout strain complemented by plasmid-borne wild-type copies of *HMT1* and *RPS2*. The *RPS2* plasmid contained a *URA3* marker, enabling counterselection against plasmid maintenance on 5-FOA plates. The *HMT1* plasmid carried *HIS3* and *CAN1* markers, allowing selection for its presence on -His plates and its loss on canavanine-containing plates in the used *can1-1* background. We then transformed this strain with a plasmid library of randomly mutagenized *rps2* alleles. Then, transformants were transferred to 5-FOA to isolate those that retained viability without the *RPS2*-containing *URA3* plasmid, thereby excluding lethal *rps2* mutations. Subsequently, we tested these *rps2* mutants for growth on canavanine-containing plates, which selected for cells that had lost the *HMT1* plasmid. While we did not identify mutants that failed to grow on these plates, which would suggest synthetic lethality of the *rps2* mutant in combination with *hmt1*Δ, we identified a mutant that grew slowly in the absence of *HMT1*, indicating a synthetic growth defect (Fig. 5*A*).

**Figure 5:**
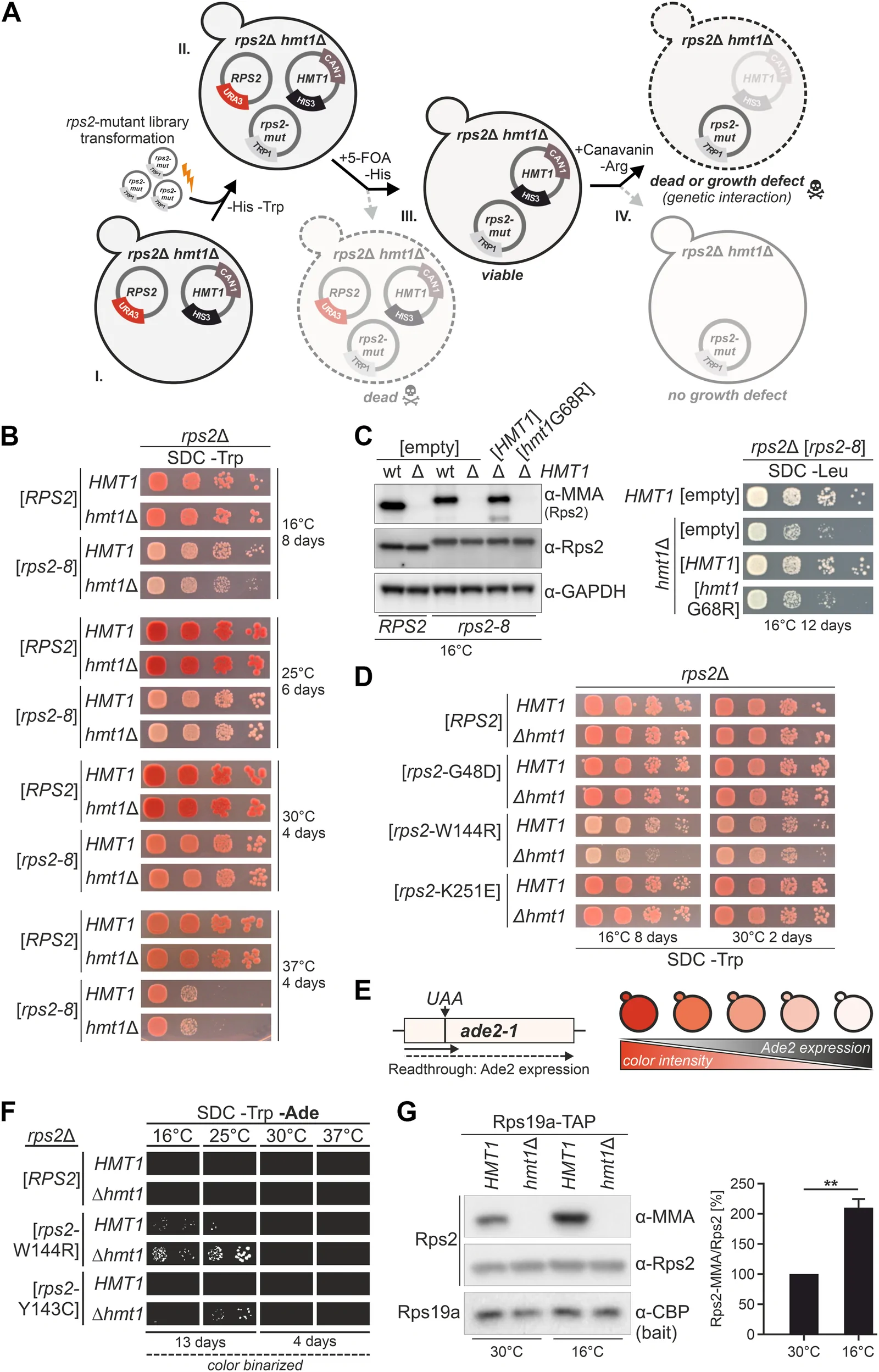
**Hmt1-dependent methylation contributes to translational fidelity of Rps2 variants at low temperatures.***A*, Schematic overview of the screen designed to identify *rps2* alleles exhibiting genetic interactions with the *hmt1*Δ null mutant. Outcomes representing undesired phenotypes are shown in transparent style. I.) The *hmt1*Δ *rps2*Δ double shuffle strain is transformed with a randomly mutagenized *rps2* library, and transformants are selected on -Trp (selection for *rps2* plasmid) and -His (selection for *HMT1* plasmid). II.) Cells are then transferred to -His plates (to select for the *HMT1* plasmid) containing 5-FOA to counterselect against the *RPS2* wild-type plasmid with the *URA3* marker. III.) Cells that fail to grow harbor lethal *rps2* alleles as they are unable to survive loss of the *URA3*-*RPS2* plasmid. Viable *rps2* mutants are streaked onto plates lacking arginine and containing canavanine. IV.) Cells retaining the *HMT1* plasmid die due to the expression of the arginine importer, encoded by *CAN1* on the same plasmid, allowing uptake of the toxic arginine analog canavanine. Cells exhibiting reduced growth upon loss of the *HMT1*-containing plasmid contain *rps2* alleles displaying a negative genetic interaction with the *hmt1*Δ null mutant. *B*, *RPS2* and *HMT1* are genetically linked at 16 °C. Cells with the indicated genotypes were, upon passage through 5-FOA containing plates, spotted onto SDC-Trp plates, which were incubated at the indicated temperatures for the indicated durations. *C*, *left*: Western blot analysis of yeast protein extracts from the indicated strains. Samples in the last four lanes correspond to the strains analyzed in the growth assay. Two *RPS2* wild-type strains (with either wild-type *HMT1* or *hmt1Δ*; lanes 1 and 2) were included as controls for Rps2 protein and methylation levels. Notably, the Rps2-8 protein migrated slightly faster than wild-type Rps2, likely due to its increased net negative charge. Wild-type *HMT1*, but not *hmt1*-G68R, rescues the synthetic growth and methylation defects. *Right*: Serial dilution growth assay of the indicated strains on SDC-Leu plates incubated at 16 °C for 12 days. *D*, the W144R substitution, but not G48D or K251E, is responsible for the *rps2-8* growth defect and its genetic interaction with *hmt1*Δ at 16 °C. Serial dilution growth assay of strains carrying the indicated *RPS2* alleles in the presence or absence of *HMT1*. Cells were spotted onto SDC-Trp plates and incubated at 16 °C or 30 °C. *E*, schematic of the *ade2-1* stop-codon readthrough assay. The *ade2-1* allele contains a premature stop codon and therefore does not produce functional Ade2, resulting in red colonies. Stop-codon readthrough restores Ade2 expression, leading to progressively lighter colony color. *F*, stop-codon readthrough assay. Growth of W303 *rps2* W144R strains carrying either wild-type *HMT1* or *hmt1*Δ on adenine-deficient medium (-Ade). Growth indicates stop-codon readthrough. The established translation fidelity mutant *rps2* Y143C served as a positive control. Images were converted to binary (*black*-and-*white*) format to enhance the visibility of faint colonies. The growth control for this experiment is shown in Figure S7*A*. *G*, Western blotting to detect levels of MMA-modified Rps2 in ribosomes tandem affinity-purified *via* Rps19a-TAP from cells grown at the indicated temperatures. Rps2 MMA signal intensities were normalized to total Rps2 levels, and mean values (± standard deviations) of three independent experiments are represented in a bar chart. For each blot, normalized Rps2 MMA levels at 30 °C were set to 100%. The same results without setting 30 °C samples to 100% are presented in Figure S7. Statistical significance was assessed by pairwise *t* test (∗∗ = *p*-value ≤ 0.01).

Sequencing of this plasmid-borne allele denoted *rps2-8*, revealed mutations leading to G48D, W144R, and K251E amino acid exchanges. The *rps2*-*8* mutant showed a growth defect, particularly at 16 and 37 °C, with the defect at 16 °C being further exacerbated by deletion of *HMT1* (Fig. 5*B*). Rps2 protein abundance and methylation levels in the *rps2-8* mutant were comparable to those in the *RPS2* wild-type strain (Fig. 5*C*, compare lanes 1 and 3) indicating that the synthetic interaction was not caused by reduced Rps2 expression of the mutant protein. To determine whether the genetic interaction was due to the loss of Hmt1’s methyltransferase activity or a structural function, we performed a complementation test with the *hmt1*-G68R allele, encoding enzymatically inactive Hmt1 (50). While wild-type *HMT1* restored Rps2 methylation and rescued the growth defect at 16 °C, *hmt1*-G68R failed to restore Rps2 methylation and did not suppress the synthetic growth phenotype (Fig. 5*C*). We conclude that the loss of Hmt1’s catalytic function in arginine methylation is responsible for the enhanced growth defect of the *rps2-8* mutant (Fig. 5*C*).

Since the *rps2-8* mutant harbors three amino acid substitutions, we next investigated whether all three changes contribute to the observed phenotype or whether a single mutation is sufficient to account for the genetic interaction with *HMT1*. We therefore generated individual mutants carrying either G48D, W144R, or K251E and analyzed their growth phenotypes alone and in combination with *HMT1* deletion. Among these variants, only W144R recapitulated the growth defect of *rps2-8* at 16 °C, which was further enhanced when *HMT1* was deleted (Fig. 5*D*). Thus, the W144R substitution is sufficient to explain the genetic interaction of the *rps2-8* mutant with *HMT1*. Because substitution of the adjacent residue Y143 has previously been shown to impair translational fidelity by increasing stop-codon readthrough and amino acid misincorporation (46), we hypothesized that the neighboring W144R substitution would have a similar effect.

Interestingly, the *rps2-8* and *rps2*-W144R strains exhibited an additional phenotype: whereas wild-type W303 colonies are red due to the *ade2-1* mutation, *rps2-8* and *rps2*-W144R colonies displayed a pink coloration, which became even lighter upon deletion of *HMT1* (Fig. 5, *B* and *D*). The *ade2-1* allele in W303 contains a premature stop codon that prevents production of functional Ade2, resulting in accumulation of red adenine biosynthesis intermediates. Importantly, *ade2* nonsense alleles are widely used as reporters for translational stop-codon readthrough, as increased readthrough restores Ade2 function and leads to colony color changes toward white (51, 52, 53) (Fig. 5*E*). Therefore, the altered colony color of r*ps2*-W144R, and its further enhancement upon *HMT1* deletion indicate increased translational readthrough in these strains. Consistent with this interpretation, the *rps2*-W144R mutant formed small colonies on adenine-deficient medium at 16 °C and 25 °C after prolonged incubation, indicating sufficient stop-codon readthrough to restore adenine biosynthesis (Figs. 5*F* and S7*A*). Strikingly, the *rps2*-W144R *hmt1*Δ strain displayed substantially stronger growth on -Ade medium, suggesting further increased readthrough efficiency in the absence of Hmt1-mediated methylation. This effect was even more pronounced than in the established translational fidelity mutant *rps2*-Y143C, for which growth on -Ade medium was only detectable upon additional deletion of *HMT1*. Together, these observations indicate that loss of Hmt1-mediated arginine methylation exacerbates translational defects of Rps2 variants with compromised fidelity, particularly at reduced growth temperatures.

Because the increased readthrough phenotype was primarily observed at 16 °C and 25 °C, and the genetic interaction between *RPS2* and *HMT1* was strongest at 16 °C, we investigated whether Rps2 methylation itself is regulated by temperature. We therefore compared Rps2 methylation levels in cells grown at 16 °C *versus* the standard growth conditions at 30 °C. Indeed, the anti-MMA antibody detected significantly higher levels of methylated Rps2 in ribosomes purified *via* Rps19a-TAP from cells grown at 16 °C (Figs. 5*G* and S7*B*). The increased methylation levels result from the activity of Hmt1, as the *hmt1*Δ strain showed no Rps2 methylation at 16 °C (Fig. 5*G*).

Together, these findings establish a genetic link between *RPS2* and *HMT1* at low temperatures and reveal that Rps2 methylation levels increase under these conditions. These observations suggest that Rps2 is not fully methylated under standard growth conditions, but that methylation can be enhanced in response to environmental cues, such as reduced growth temperature. Increased Rps2 methylation may therefore represent a mechanism to maintain translational fidelity and ribosome function under conditions that challenge protein synthesis.

## Discussion

In this study, we investigated the interplay between importin binding, chaperone interaction, and arginine methylation in the N-terminal unstructured extension of Rps2. This N-terminal extension is conserved among eukaryotes and has recently been shown to prevent mistargeting to mitochondria (54). Given its role as a nuclear localization element, we propose that efficient recognition of this sequence by the nuclear import machinery contributes to correct Rps2 localization and thereby prevents mitochondrial mislocalization.

As Tsr4 and Pse1 recognize overlapping regions within this Rps2 N-terminal extension, we investigated whether these factors can simultaneously associate with this Rps2 element or whether they compete for binding. Our data demonstrate that Tsr4, Pse1, and Rps2(5–28) do not form a stable ternary complex. Instead, FP experiments show that Tsr4 efficiently displaces Pse1 from the shared binding site, supporting competitive binding.

Using mutagenesis experiments based on AlphaFold three structural predictions, we identified R11 and R17 within the Rps2 N-terminal extension as critical residues for Pse1 binding and N-terminal NLS function. Although these residues do not strictly match the previously proposed Pse1-specific IK-NLS motif (48), they share physicochemical features with this motif, as it also contains two positively charged residues. Notably, R11 has been reported in three independent studies, and R17 in one study, as a site of arginine monomethylation and asymmetric dimethylation by Hmt1 (39, 40, 41). While we readily detected methylated R11 using an anti-MMA antibody, we were unable to confirm R17 modification by western blotting. This may reflect condition-specific R17 methylation or, alternatively, limitations of antibody recognition, as the tested anti-MMA and anti-DMA antibodies were generated against methylated arginine residues within RGG motifs and may therefore not efficiently detect modifications outside this context. Because R11 methylation was robustly detectable, we focused on investigating its potential impact on Rps2 interactions.

R11 dimethylation modestly reduced Pse1 binding to an Rps2(5–28) peptide (Fig. 3*C*). This observation is consistent with previous reports that arginine methylation of importin cargoes, including FUS and PABPN1, decreases their affinity for the human importin transportin 1 (55, 56). In contrast, Tsr4 binding to the Rps2(5–28) peptide was largely unaffected by R11 methylation.

To understand the potential functional implications of reduced Pse1 binding upon Rps2 R11 methylation, it is important to consider the timing of this modification during the Rps2 life cycle. We found that Rps2 is already methylated in complexes containing its dedicated chaperone Tsr4. Since Tsr4 is thought to associate with newly synthesized Rps2 in the cytoplasm and to be released prior to nuclear import (38), these findings indicate that Rps2 methylation occurs before importin-mediated nuclear import (Fig. 2*C*).

The functional consequences of reduced Pse1 affinity for the methylated N-terminal extension are likely influenced by the more complex binding environment of full-length Rps2. In addition to the N-terminal nuclear localization element, Rps2 contains a second, internal Pse1-binding region encompassing residues 95 to 99. Thus, Pse1 binding to full-length Rps2 may be stabilized through simultaneous engagement of both interaction sites, resulting in a higher overall affinity than observed for the isolated N-terminal extension. Moreover, although Tsr4, Pse1, and the isolated Rps2(5–28) peptide do not form a stable ternary complex, a configuration in which Tsr4 remains associated with the N-terminal extension while Pse1 engages the internal binding site cannot be excluded for full-length Rps2.

Our Y2H competition assay likely underestimates Pse1 competition with Tsr4, as importin-cargo interactions are transient in the nucleus due to RanGTP-mediated cargo release. Nevertheless, weakening Tsr4 binding using the Tsr4-6 variant revealed that Pse1 overexpression reduces Tsr4-Rps2 interaction, particularly in the absence of Hmt1. Although we did not detect a significant effect of R11 methylation on competition *in vitro*, the observed competition in the context of full length Rps2 and in cells supports a hypothesis in which R11 methylation weakens Pse1 binding to the Rps2 N-terminal extension and thereby shifts the equilibrium toward Tsr4 association. In the context of full-length Rps2, such methylation-dependent modulation could therefore favor a Tsr4-Rps2-Pse1 intermediate in which Tsr4 protects the N-terminal extension while Pse1 remains associated through its internal binding site. Future structural and biochemical studies, including reconstitution of full-length Rps2–Tsr4–Pse1 complexes, will be required to test this model and define how R11 methylation influences Pse1 engagement of the distinct Rps2 binding sites.

Interestingly, Rps2 methylation increases at low temperature. Whether this reflects a general adaptation affecting multiple methylated proteins or a specific regulatory mechanism for Rps2 remains unclear. Increased methylation could favor stabilization of a Tsr4-Rps2 or a Tsr4-Rps2-Pse1 intermediate, which may become particularly relevant when ribosome assembly is slowed under cold conditions. Following Tsr4 release by an as-yet unidentified mechanism, Pse1 may then engage both Rps2 binding sites to promote nuclear import.

Although Rps2 is methylated before nuclear import, this modification may also affect later steps of the Rps2 life cycle. For example, methylation of the Rps2 N-terminal extension could facilitate efficient cargo-importin dissociation in the nucleus by weakening Pse1 interaction. Moreover, we identified a downstream consequence of impaired methylation linked to the role of Rps2 in maintaining translational fidelity. Blocking arginine methylation further increased translational inaccuracies in *rps2* mutants with compromised translation fidelity. Whether this phenotype results from altered Rps2 trafficking and reduced incorporation into mature ribosomes or reflects a direct effect of methylation on Rps2 function within the ribosome, remains to be determined. Nevertheless, these findings demonstrate that arginine methylation contributes to the functional regulation of Rps2.

Notably, although our study was performed in *S. cerevisiae*, the conservation of key components provides a framework for investigating RPS2 methylation in higher eukaryotes. Human RPS2 is recognized by the Tsr4 ortholog PDCD2 and is also methylated within its N-terminal extension (57, 58, 59). However, in mammals this modification is catalyzed by PRMT3, which, unlike yeast Hmt1, has an additional structural role in RPS2 assembly. PRMT3 forms a stable complex with RPS2 and phenotypes caused by PRMT3 depletion cannot be fully reproduced by catalytically inactive mutants, indicating that loss of its structural function contributes substantially to these defects (59, 60, 61, 62, 63).

The absence of an analogous structural role for Hmt1 allowed us to dissect the specific contribution of Rps2 methylation independent of methyltransferase-mediated chaperone activity. Our findings reveal a functional role for Rps2 methylation in the early life of Rps2 and provide a basis for future studies investigating how this modification contributes to ribosome assembly and function in mammalian systems.

## Experimental procedures

### Yeast strains and genetic methods

All *S*. *cerevisiae* strains employed in this study are listed in Table S1 and were generated using established protocols for chromosomal deletion and tagging (64, 65). *E*. *coli* strains are also included in Table S1. Plasmid constructs were generated using standard recombinant DNA methodologies and are listed in Table S2. The integrity of all PCR-amplified DNA fragments was confirmed by sequencing.

The Rps19a-TAP *RPS2* shuffle and Rps19a-TAP *RPS2* shuffle *hmt1*Δ strains were transformed with the pRS315-*RPS2* or pRS315-*rps2* R11K plasmid. Following transformation, cells were streaked onto plates containing 5-FOA (Thermo Scientific) to counterselect against the pRS316-*RPS2* (*URA3*) shuffle plasmid.

The *rps2* and *tsr4* mutants utilized in this study were generated by random PCR mutagenesis, using a protocol previously described (10). The pRS315-*tsr4-6* plasmid was then used as a template for cloning of the pG4ADC111-*tsr4-6* plasmid.

### Screening for *rps2* alleles that cause genetic interactions in combination with *HMT1* deletion

To screen for *rps2* alleles that cause synthetic growth defects in combination with *hmt1*Δ, an *rps2*Δ *hmt1*Δ double shuffle strain carrying a *can1-1* mutation and harboring two counterselectable plasmids was used: while the pRS316-*RPS2* plasmid allowed for selection on -Ura media and counterselection on 5-FOA-containing plates, the pHT-*HIS3*-*CAN1*ΔCEN-*HMT1* plasmid allowed for selection on -His media and counterselection on canavanine-containing plates. First, an *rps2* mutant library (*TRP1*) was generated by random PCR mutagenesis as previously described (10) and transformed into this shuffle strain. Transformants were streaked onto selective plates lacking tryptophan and histidine (-Trp -His) to maintain the *rps2* mutant plasmid and the *HMT1* plasmid. Subsequently, cells were transferred to plates containing 5-FOA (1 g/L) and lacking histidine and incubated at 30 °C, to select for cells that lost the pRS316-*RPS2* (*URA3*) plasmid but retained the pHT-*HIS3*-*CAN1*ΔCEN-*HMT1* plasmid. Hence, all *rps2* mutants that are viable in the presence of *HMT1* were selected in this step. Next, cells were transferred to non-selective yeast extract peptone dextrose (YPD) media to allow for the loss of the pHT-*HIS3*-*CAN1*ΔCEN-*HMT1* plasmid. Following this, colonies were transferred to media containing canavanine (60 mg/L) and lacking arginine to promote the uptake of the toxic canavanine in the presence of the *CAN1*-containing plasmid and consequently select for cells that lost the pHT-*HIS3*-*CAN1*ΔCEN-*HMT1* plasmid. At this stage, cells exhibiting severe growth defects were identified and, thus, potential *rps2* mutants with synergistic growth defects in combination with *HMT1* deletion. Plasmids were isolated from these cells and subsequently transformed into both an *RPS2* shuffle strain and an *RPS2* shuffle *hmt1*Δ strain to reproduce and verify the observed growth phenotypes.

### Rps2 N-terminal peptides

Synthetic peptides corresponding to amino acids 5 to 28 of Rps2, followed by a C-terminal lysine residue conjugated with a Cy5 fluorophore *via* its sidechain ε-amino group, were obtained in three different modification states of arginine 11 - non-methylated, monomethylated, or asymmetrically demethylated—from PSL Peptide Specialty Laboratories GmbH, Heidelberg, Germany. Lyophilized peptides were stored at −20 °C in the dark and dissolved before use in 50 mM Tris-HCl (pH 7.7), 150 mM NaCl, 10 mM MgCl_2_, 0.05% Tween 20, 0.5 mM dithiothreitol (DTT) for dot blot and fluorescence intensity change assays or in 20 mM Tris-HCl (pH 7.7), 200 mM NaCl, 0.5 mM DTT for analytical size exclusion chromatography.

### Preparation of yeast protein extracts, dot blot assay, and western blotting

Total yeast protein extracts were prepared as described previously (66). Cultures were grown to an OD_600_ of around 0.5, and protein extracts were prepared from an equivalent of one OD_600_ of cells.

For dot blot analysis, 5 μl of each Rps2 peptide solution, starting from a concentration of 200 μM, was spotted onto a PVDF membrane in serial 1:5 dilution steps. Peptide loading was confirmed by detection of the Cy5 fluorophore using a ChemiDoc™ Touch Imaging System (Bio-Rad). The methylation state of the peptides was tested using an anti-MMA antibody (1:2000, Cell Signaling Technology, Leiden, Netherlands, cat. no. 8711), an anti-MMA/DMA antibody (1:2000, Abcam, Amsterdam, Netherlands, cat. no. Ab412), two anti-ADMA antibodies (1:2000, Merck-Millipore, cat. no. ASYM24 and 1:2000, Cell Signaling Technology, cat. no. 13522).

Protein samples for Western blot analyses were separated on NuPage™ 4 to 12% Bis-Tris gels (Invitrogen) followed by blotting on PVDF membranes. Specific protein detection was performed using the following primary rabbit antibodies: anti-CBP (1:5000; Merck-Millipore, cat. no. 07-482), anti-MMA (1:1000, Cell Signaling Technology, cat. no. 8711), anti-Tsr4 (custom-made against recombinant Tsr4, Eurogentec) anti-Rps2/Rpl30 (1:2000; kindly provided by Jonathan Warner), anti-Rpl16 (1:15,000; kindly provided by Sabine Rospert). The specificity of the anti-Tsr4 and anti-Rps2 antibodies was validated by comparing cell lysates from untagged and Flag-tagged strains. A mobility shift of the detected band in the Flag-tagged strain confirmed that the antibodies recognized the correct proteins. Validation of the anti-MMA antibody is shown in Figure 2*B*. Notably, the MMA signal was abolished upon deletion of *HMT1*, confirming the specificity of the antibody under the experimental conditions used. The anti-CBP antibody was validated in Figure 2, *B*–*D* by the reduced molecular weight of the proteins detected in the eluates compared to the lysates, reflecting cleavage of the TAP tag by TEV protease. The commercial anti-GAPDH antibody was validated by the manufacturer.

For detection of all primary antibodies used in the dot blot assay and western blotting, a horseradish peroxidase-conjugated anti-rabbit secondary antibody was used (1:15,000; Sigma-Aldrich, cat. no. A0545). Signals were visualized using the Clarity Western ECL Substrate Kit (Bio-Rad) and imaged using the ChemiDocTM Touch Imaging System (Bio-Rad). Signals were quantified using Image Lab (Bio-Rad).

### Tandem affinity purification (TAP)

Cells were grown in 4 L YPD to an optical density (OD_600_) of two at 30 °C. TAP purifications were performed in a buffer containing 50 mM Tris-HCl (pH 7.5), 100 mM NaCl, 1.5 mM MgCl_2_, 0.1% NP-40, 1 mM DTT, and 1x Protease Inhibitor Mix FY (Serva Electrophoresis GmbH). Cells were lysed by mechanical disruption using glass beads and cell lysates were incubated with 300 μl IgG Sepharose™ 6 Fast Flow (GE Healthcare) for 60 min at 4 °C. After incubation, the IgG Sepharose beads were transferred into Mobicol columns (MoBiTec, Göttingen, Germany) and washed with 10 ml buffer. Then, 10 μl of TEV protease [1 mg/ml] was added and elution from the beads was performed under rotation for 90 min at room temperature. After the addition of 2 mM CaCl_2_, TEV eluates were incubated with 300 μl Calmodulin Sepharose™ 4B beads (GE Healthcare) for 60 min at 4 °C. After washing with 5 ml buffer containing 2 mM CaCl_2_, proteins were eluted from Calmodulin Sepharose beads with elution buffer consisting of 10 mM Tris-HCl (pH 8.0), 5 mM EGTA, and 50 mM NaCl under rotation for 20 min at room temperature. Finally, the eluates were subjected to further analysis by western blotting using specific antibodies.

### Purification of recombinant Tsr4

*TSR4*-(GA)3-His6x was cloned into the pCOLADuet-1 vector (Novagen), enabling the expression of C-terminally (GA)3-His6x-tagged Tsr4. The plasmid was transformed into *E*. *coli* BL21 CodonPlus(DE3)-RIL cells (Novagen). Transformants were grown in 1 L ZY autoinduction medium (described in (67)) containing 50 μg/ml kanamycin and 50 μg/ml chloramphenicol at 37 °C until reaching an OD_600_ of 0.5. Cultures were then shifted to 16 °C and incubated for 20–30 h to enable autoinduction of protein expression *via* lactose following glucose depletion.

Cell pellets were resuspended in 1.5 volumes of lysis buffer containing 50 mM Tris–HCl (pH 7.7), 500 mM NaCl, 1 mM DTT, 1x HP protease inhibitor mix (Serva), 1 mM phenylmethylsulphonyl fluoride (PMSF), and 1 mg/ml lysozyme, and cells were lysed by sonication. Cell lysates were centrifuged at 44,000*g* at 4 °C for 45 min to remove insoluble material. Then, imidazole was added to the supernatant to a final concentration of 50 mM. Subsequently, the cleared lysate was loaded onto a 1-ml HisTrap™ HP column (Cytiva). The column was run on an ÄKTA FPLC system (Cytiva) at 1 ml/min flow rate at 4 °C in buffer containing 50 mM Tris–HCl (pH 7.7), 500 mM NaCl, 0.5 mM DTT. (His)6-tagged Tsr4 was eluted using a linear imidazole gradient (25 ml, 50–500 mM) at 0.5 ml/min. Fractions containing Tsr4 were pooled and concentrated to a volume of 1 ml using Amicon Ultra-4 Centrifugal Filter Units 30k (Merck Millipore).

The concentrated Tsr4 was further purified by size exclusion chromatography on a Superdex 200 Increase 10/300 Gl column (GE Healthcare) operated on an ÄKTA FPLC system at a flow rate of 0.5 ml/min at 4 °C in buffer containing 20 mM Tris–HCl (pH 7.7), 200 mM NaCl, 0.5 mM DTT. Tsr4-containing fractions were pooled, concentrated as described above, and flash-frozen in liquid nitrogen for storage at −80 °C.

### Purification of recombinant Pse1

N-terminally GST-tagged Pse1 was expressed from a pGEX-4TEV-*PSE1* plasmid in *E. coli* CodonPlus(DE3)-RIL cells (Novagen). Cells were cultured in 1 L ZY-medium containing 100 μg/ml ampicillin and 50 μg/ml chloramphenicol at 37 °C to an OD_600_ of 0.5. The cultures were incubated at 16 °C for 20 to 30 h. Protein expression was induced with lactose-containing ZY medium after glucose was depleted.

Cell pellets were resuspended in 1.5 volumes of lysis buffer containing 50 mM Tris–HCl (pH 7.7), 500 mM NaCl, 1 mM DTT, 1× HP protease inhibitor mix (Serva), 1 mM PMSF, and 1 mg/ml lysozyme, and cells were lysed by sonication. Cell lysates were centrifuged at 44,000*g* at 4 °C for 45 min to remove insoluble material. Next, cleared lysate was incubated for 2 h under rotation at 4 °C with Glutathione Agarose beads (Merck-Millipore). Beads were washed three times with buffer and then incubated overnight with TEV protease at 4 °C to elute Pse1. The eluate was concentrated to a volume of 1 ml using Amicon Ultra-4 Centrifugal Filter Units 30k (Merck Millipore).

Next, Pse1 was further purified by size exclusion chromatography on a Superdex 200 Increase 10/300 Gl column (GE Healthcare) operated on an ÄKTA FPLC system at 0.5 ml/min flow rate at 4 °C in buffer containing 20 mM Tris–HCl (pH 7.7), 200 mM NaCl, 0.5 mM DTT. Pse1-containing fractions were pooled, concentrated as described above, and flash-frozen in liquid nitrogen for storage at −80 °C.

### Protein interaction measurement by ligand-induced change in initial fluorescence

150 μM Tsr4 and 45 μM Pse1 stock solutions were prepared in assay buffer containing 50 mM Tris-HCl (pH 7.7), 150 mM NaCl, 10 mM MgCl_2_, 0.05% Tween20 and 0.5 mM DTT. From these, 14 step serial dilutions, each with a 1:1 fold dilution per step, were prepared in assay buffer, with a final volume of 6 μl in each vial. To each vial, 6 μl of Cy5-labeled Rps2 peptide, dissolved in assay buffer, was added, resulting in a final peptide concentration of 10 nM. Reactions were incubated for 5 min at room temperature and then transferred into Monolith premium capillaries (Nanotemper). Fluorescence intensity was measured using a Monolith NT.115 instrument (Nanotemper). The experiment was performed in triplicate, using independently purified protein batches of Tsr4 or Pse1. Dose-response curves were generated and analyzed to determine dissociation constants (*K*_*d*_ values) using MO.AffinityAnalysis software (Nanotemper).

*K*_*d*_ values from three binding measurements, each performed using independently purified Pse1 and Tsr4, were used to calculate means and standard deviations. A one-way ANOVA, followed by Dunnett’s multiple comparison test, was performed to assess significance using GraphPad Prism 10.

### Fluorescence polarization-based binding assays

To quantify affinities, we added increasing concentrations of Tsr4 (0–1500 nM, final concentrations) or Pse1 (0–2000 nM, final concentrations) to 5 nM Cy5-labeled Rps2(5–28) in binding buffer (150 mM NaCl, 50 mM Tris-HCl, pH 7.7, 10 mM MgCl_2_, 0.05% Tween-20, 0.5 mM DTT) and incubated the mixtures for 30 min at room temperature. To assess competition between Tsr4 and Pse1 for peptide binding, we incubated unmethylated or dimethylated Rps2(5–28)-Cy5 (5 nM) with Pse1 (2000 nM) for 30 min at room temperature, then added increasing concentrations of Tsr4 (0–2000 nM, final concentrations) and incubated for an additional 30 min at room temperature. We measured fluorescence polarization in 384-well plates using a Spark multimode microplate reader (Tecan Group Ltd, Männedorf, Switzerland) at 25 °C, with excitation at 600 nm and emission detected at 670 nm.

To determine affinities, we plotted baseline-corrected changes in fluorescence polarization against protein concentration and fit the data to a one-site binding model (A = Amax x/(Kd + x); Amax, fitted maximum of peptide bound; x, protein concentration; Kd, dissociation constant) using OriginPro 2025 (OriginLab Corporation, Northampton, MA). To quantify binding competition, we fit baseline-corrected changes in fluorescence polarization to a single-exponential decay function (A = AΔ(exp(−x/c)) + Amin; AΔ, anisotropy span; x, protein concentration; c, decay constant; Amin, plateau) using OriginPro 2025.

### Fluorescence microscopy

Yeast strains were cultured at 30 °C in SDC medium lacking leucine (SDC–Leu) until reaching an OD_600_ of approximately 0.5. Fluorescence microscopy was performed using a Leica DM6 B microscope equipped with a DFC 9000 GT camera, a PLAN APO 100× objective, narrow-band GFP or RHOD ET filters, and LasX software. Rps2 fragments and their variants were expressed as C-terminally 3xyEGFP-tagged fusion proteins under the control of the *ADH1* promoter from a centromeric *LEU2* plasmid. These plasmids were transformed into a W303 *ade3*Δ strain expressing Nop58-yEmCherry. Given that *RPS2* is essential, the localization of the various Rps2-3xyEGFP fusion proteins was examined in strains carrying the chromosomal wild-type *RPS2* gene.

### Yeast two-hybrid assays

Protein-protein interactions between Rps2 (including fragments and variants thereof) and either Pse1(302–1089) or Tsr4 were assessed using yeast two-hybrid (Y2H) assays in the reporter strains PJ69-4A or PJ69-4A *hmt1*Δ. These strains enable detection of both weak (*via* the *HIS3* reporter) and strong (*via* the *ADE2* reporter) interactions. Two plasmids were co-transformed into PJ69-4A or PJ69-4A *hmt1*Δ; one encoding the bait protein fused to the Gal4 DNA-binding domain (G4BD; *TRP1* marker) and the other one encoding the prey protein fused to the Gal4 activation domain (G4AD; *LEU2* marker). For competition Y2H experiments, high-copy plasmids (*URA3* marker) encoding Pse1 or Tsr4 as competitors were additionally transformed.

After the selection of transformants on plates lacking leucine and tryptophan (SDC-Leu-Trp) or lacking leucine, tryptophan, and uracil (SDC-Leu-Trp-Ura), cells were spotted onto -Leu-Trp or -Leu-Trp-Ura plates as well as onto plates lacking histidine, leucine, and tryptophan (SDC-His-Leu-Trp) or lacking histidine, leucine, tryptophan, and uracil (SDC-His-Leu-Trp-Ura), and lacking adenine, leucine, and tryptophan (SDC-Ade-Leu-Trp) or lacking adenine, leucine, tryptophan, and uracil (SDC-His-Leu-Trp-Ura), respectively. Plates were incubated for 3 days at 30 °C.

### Analytical size exclusion chromatography

To assess interactions between Rps2 peptides and Tsr4 or Pse1, all components were mixed in equimolar amounts (300 μM each) in buffer containing 20 mM Tris–HCl (pH 7.7), 200 mM NaCl, 0.5 mM DTT. In a separate condition, a 10-fold molar excess of Rps2 peptide (3 mM) was included. After incubation for 10 min at room temperature, 50 μl of each sample was loaded onto a Superdex 200 3.2/300 size exclusion column (GE Healthcare). Tsr4 and Pse1 were detected by monitoring absorbance at 280 nm, while Cy5-labeled Rps2 peptides were detected by fluorescence at 630 nm.

## Data availability

All study data are included within the manuscript.

## Supporting information

This article contains supporting information (7, 10, 37, 54, 68, 69, 70, 71, 72).

## Conflict of interest

The authors declare that they have no conflicts of interest with the contents of this article.
