# Supplementary material for: The N-terminal extension of ribosomal protein Rps2 shapes its early fate through Chaperone–Importin competition and arginine methylation
Source: J Biol Chem. 2026 Aug 25;302(10):113481. doi: 10.1016/j.jbc.2026.113481 (PMC13627120; doi:10.1016/j.jbc.2026.113481)
Supplement: Supplementary file 1 [file mmc1.pdf]

Supporting Information 1 - Supporting Figures

The N-terminal Extension of Ribosomal Protein Rps2 Shapes Its Early Fate through Chaperone–Importin Competition and Arginine Methylation

Maximilian Mack[1,2], Benjamin Pillet[3], Nicole Holton[4], Ingrid Zierler[1,2], Stephanie Himpich[4], Phyllis Jessen[1,2], Markus C. Wahl[4,5], Dieter Kressler[3], Brigitte Pertschy[1,2,*]

Figure S1
Figure S2
Figure S3
Figure S4
Figure S5
Figure S6
Figure S7

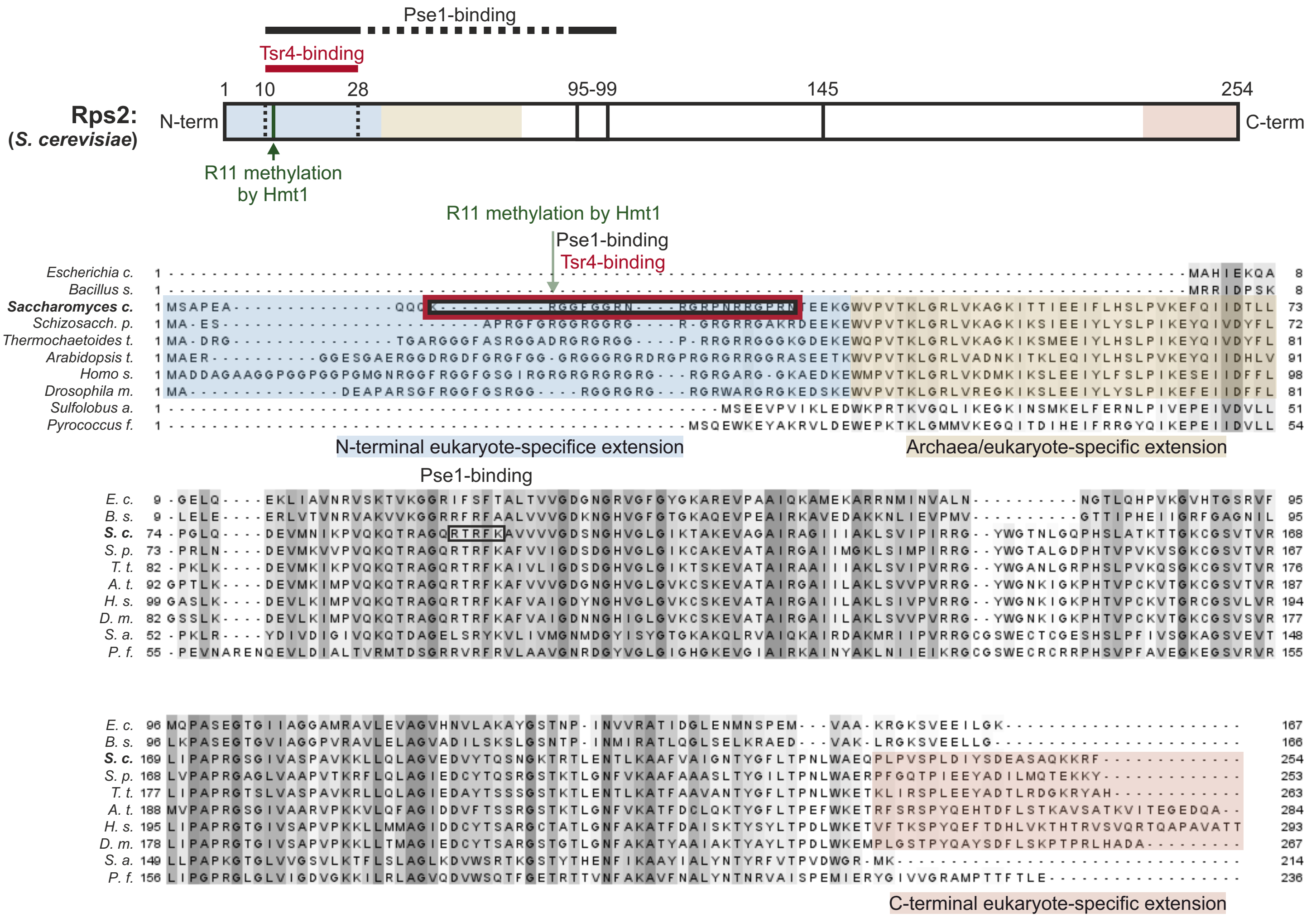

Figure S1: Schematic overview and sequence alignment of Rps2. The sequence alignment highlights the conserved and unique regions of Rps2 across species. The eukaryote-specific N-terminal extension is marked in blue, while an N-terminal segment shared by eukaryotes and archaea but missing in bacteria is shown in yellow. The eukaryote-specific C-terminal extension is highlighted in red. Annotations are adopted from Oborská-Oplová et al. 2025 (54). Binding sites of Pse1 and Tsr4 are indicated by black and red boxes, respectively. Sequences were aligned using Clustal Omega and visualized in Jalview (68).

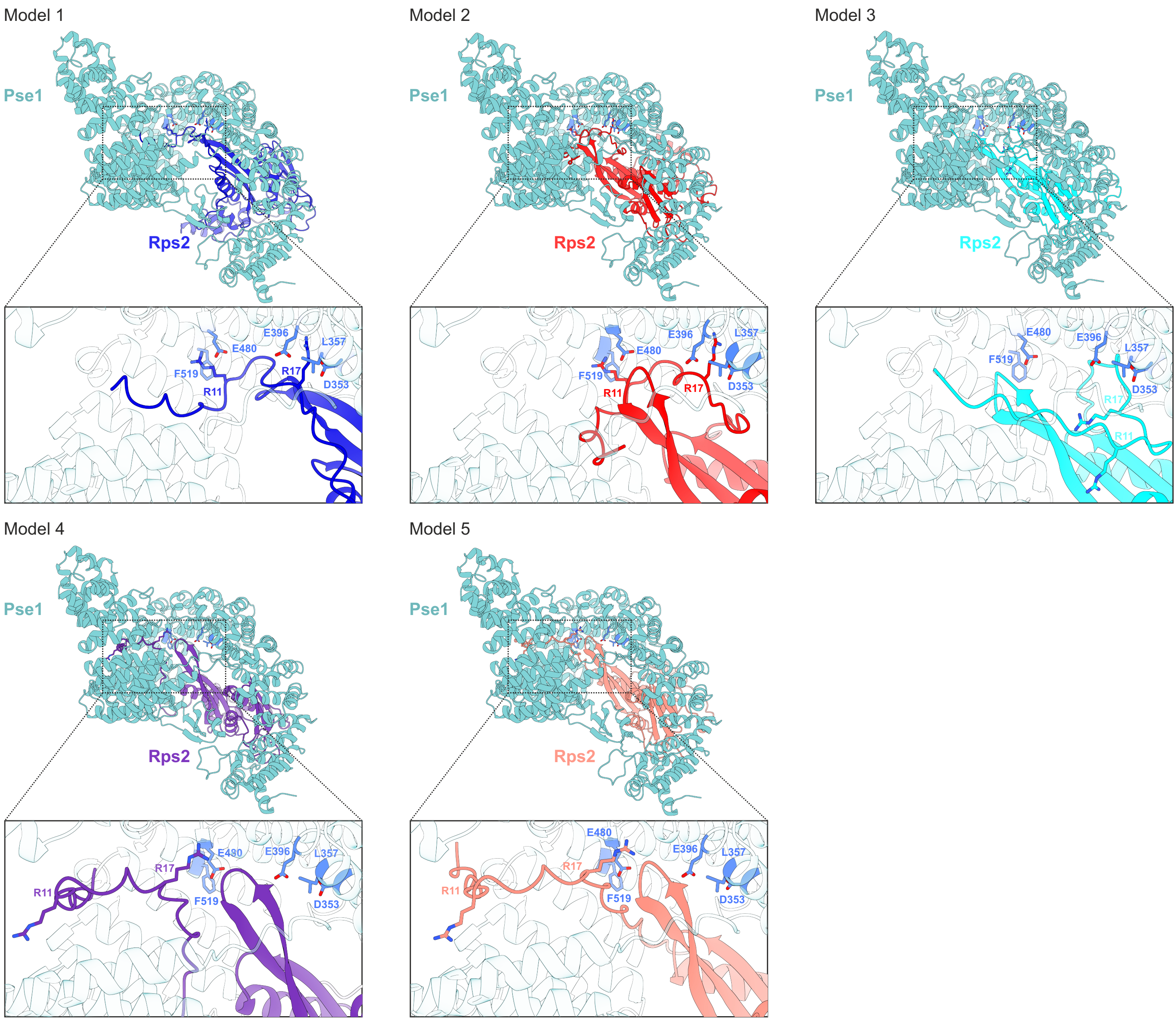

Figure S2: Top five-ranked AlphaFold 3 Pse1-Rps2 models. All top five models are represented from the same view with respect to Pse1, and sections of Rps2's N-terminal region potentially interacting with the canonical NLS-binding site of Pse1 are zoomed in.

A

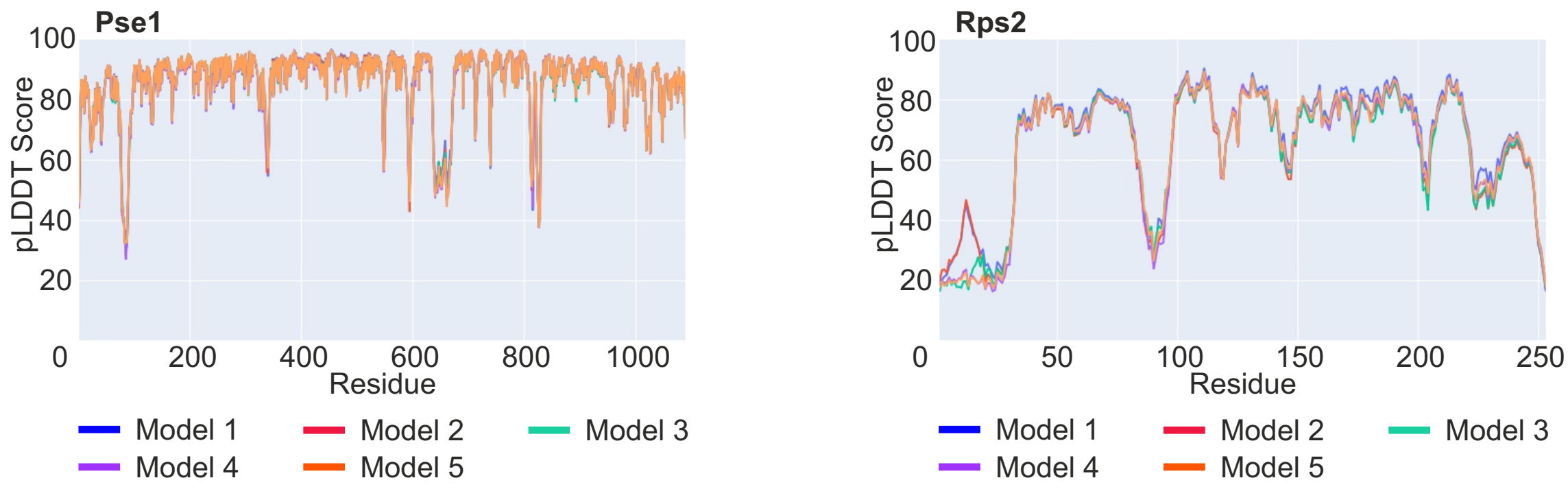

B

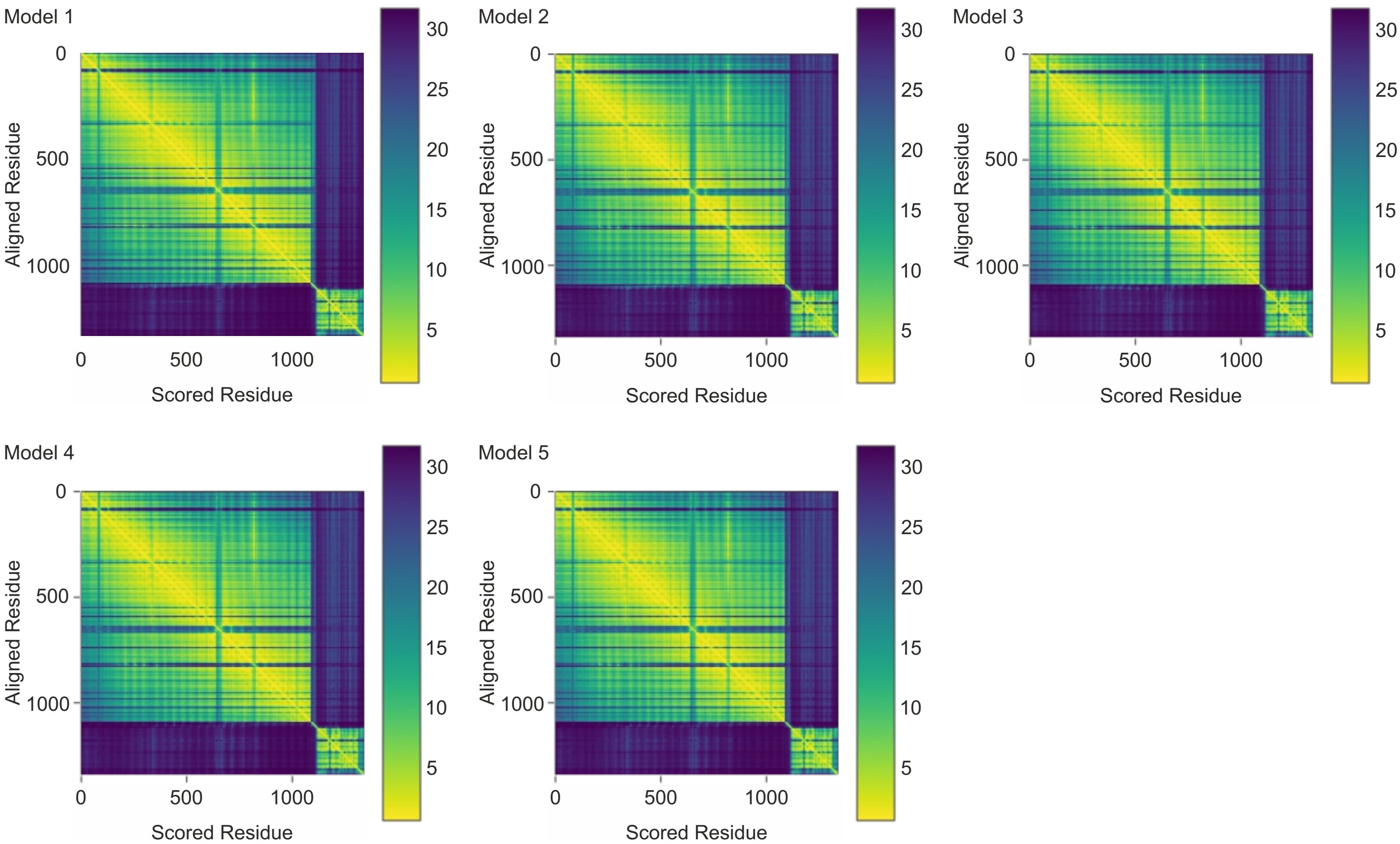

C

Model	Ranking Score	ipTM	pTM
Model 1	0.38	0.28	0.72
Model 2	0.37	0.27	0.72
Model 3	0.34	0.23	0.72
Model 4	0.33	0.22	0.72
Model 5	0.33	0.22	0.72

Figure S3: Confidence metrics for the AlphaFold 3 models shown in Figure S2. **(A)** Per-residue confidence scores, shown as predicted Local Distance Difference Test (pLDDT) values, for all predicted Pse1 (left) and Rps2 (right) models. pLDDT scores range from 0 to 100, with higher values indicating greater confidence in the local structural prediction. **(B)** Predicted Aligned Error (PAE) plot showing the expected positional error between all residue pairs in the predicted complex. Lower PAE values indicate higher confidence in the relative positioning of domains and the predicted protein-protein interface. **(C)** Global confidence metrics for the predicted complex, including the predicted TM-score (pTM), interface predicted TM-score (ipTM), and the overall ranking confidence score calculated by AlphaFold. Higher pTM and ipTM scores indicate greater confidence in the overall fold and the predicted molecular interface, respectively.

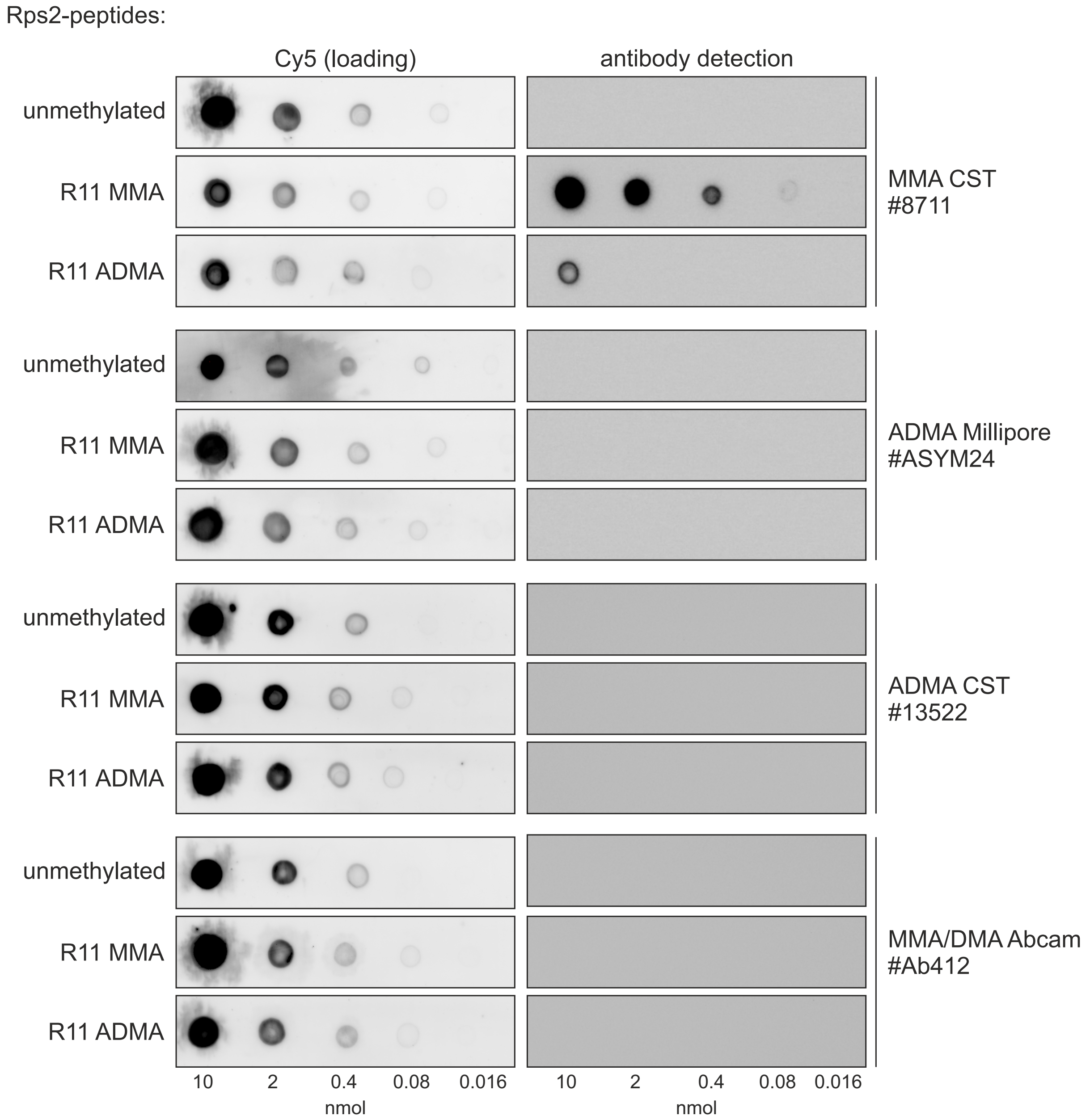

Figure S4: Dot blots of Rps2(5-28) peptides for antibody testing. Cy5-labeled peptides corresponding to Rps2(5-28) with different R11 methylation states were spotted onto a PVDF membrane at the indicated amounts. Left panels: Cy5 fluorescence was detected as control for peptide loading. Right panels: Methylation was detected using the indicated anti-arginine methylation antibodies. Note that the identical dot blot analysis shown in Figure 2A is reproduced as the uppermost panel to facilitate comparison with the results obtained with the other antibodies.

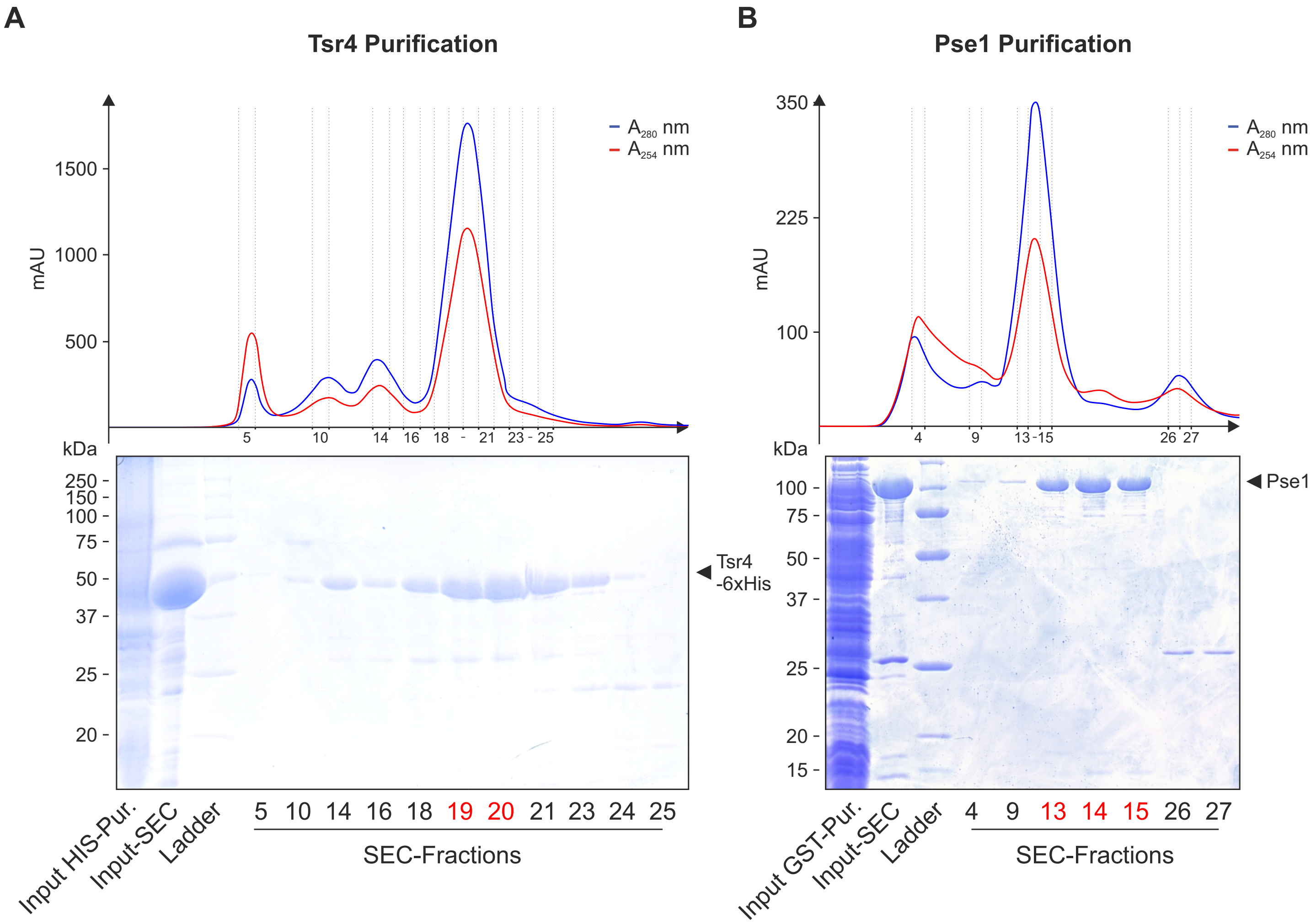

Figure S5: Examples of SEC profiles and Coomassie-stained gels of purifications from recombinantly expressed Tsr4-(His)6 (A) and Pse1 (B) to demonstrate the purity of the used proteins. Fractions highlighted in red font color were pooled, concentrated, and used for interaction assays.

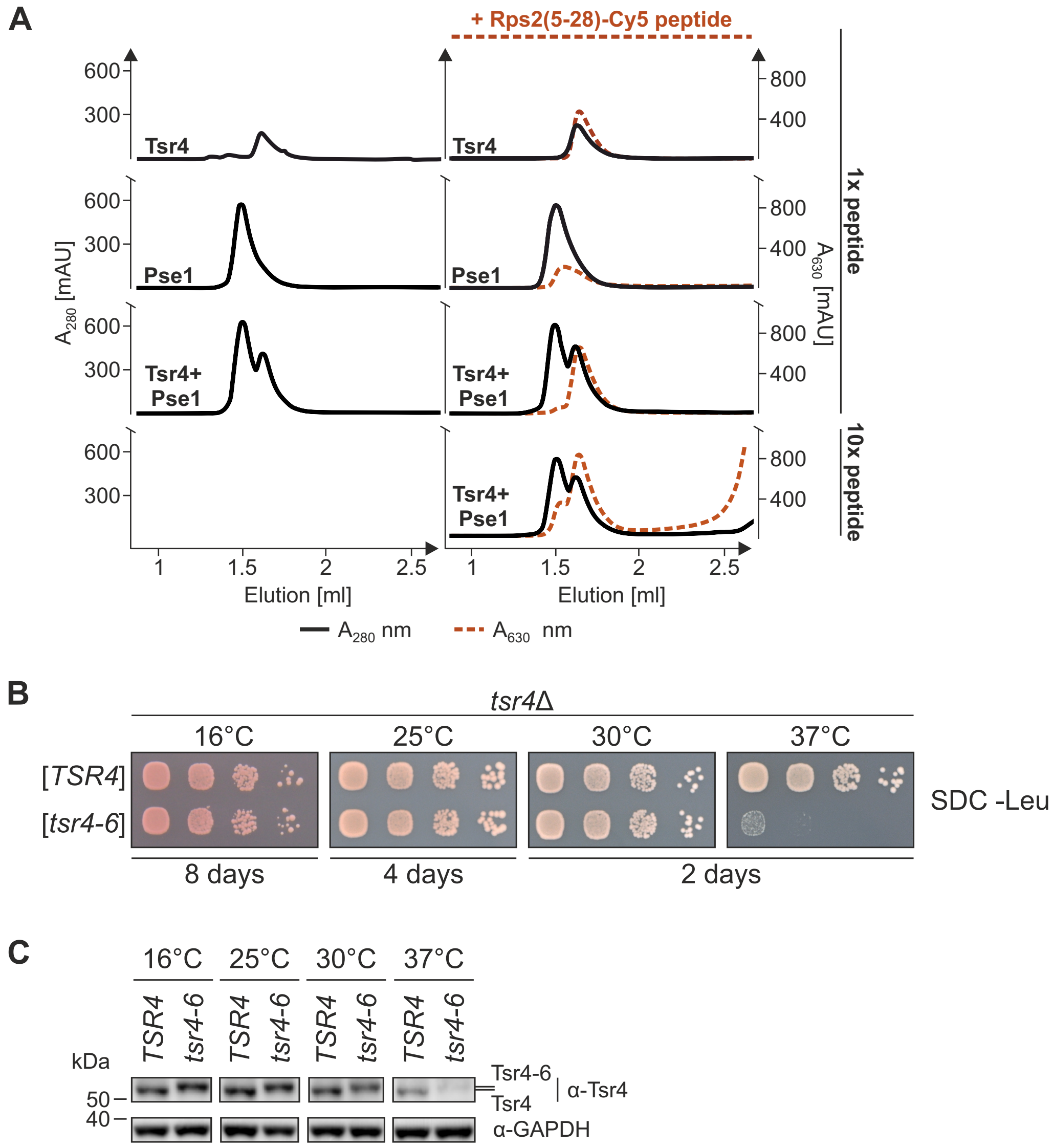

Figure S6: **Pse1, Tsr4, and Rps2(5-28) do not form a ternary complex. (A)** Analytical size exclusion chromatography of Tsr4, Pse1, and Cy5-labeled Rps2(5-28). Tsr4 and Pse1 protein elution was monitored by absorbance at 280 nm (black lines), and the Cy5-labeled Rps2 peptide by absorbance at 630 nm (red dashed lines). Left: Elution profiles of Tsr4 and Pse1 in the absence of Rps2 peptide. Right: Elution profiles of Tsr4 and Pse1 in the presence of Cy5-labeled Rps2(5-28). The first three rows display experiments with equimolar amounts of Tsr4, Pse1, and Rps2 peptide. The fourth row in the right panel shows results with a 10-fold molar excess of Rps2 peptide. **(B)** Growth phenotype of the *tsr4-6* mutant. *tsr4* cells complemented with plasmids with either wild-type *TSR4* or the *tsr4-6* allele were spotted in serial dilutions on SDC-Leu plates and incubated for the indicated number of days. **(C)** Tsr4-6 protein levels are reduced at elevated temperatures. Total yeast protein extracts were prepared from the strains shown in (B) after growth at the indicated temperatures and analyzed by western blotting to evaluate Tsr4 protein levels. GAPDH served as loading control.

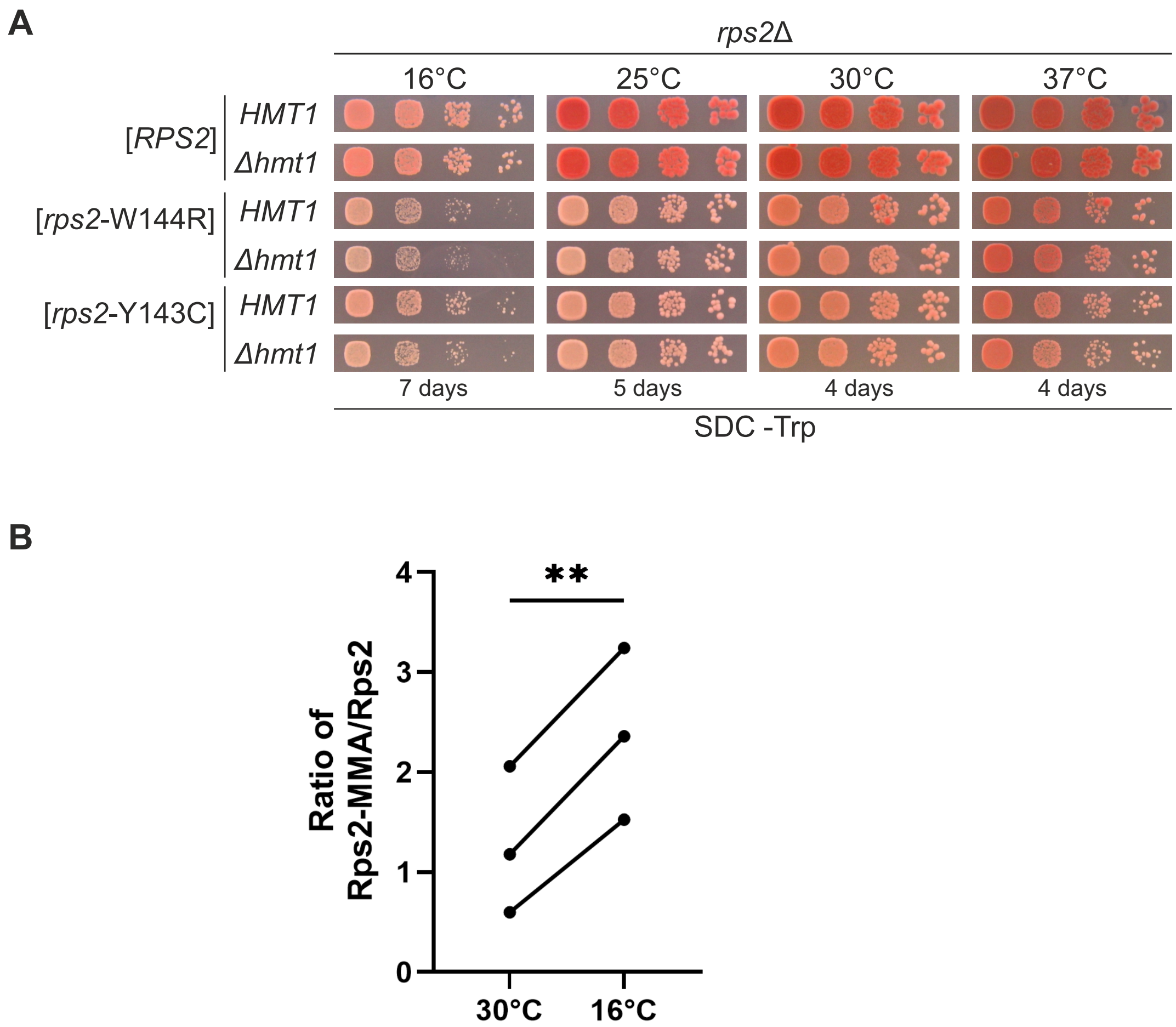

Figure S7: **The role of Rps2 methylation at low temperatures. (A)** Growth control for the ADE2 readthrough assay shown in Figure 5F. **(B)** Quantification of Rps2 R11 methylation normalized to Rps2 amounts of the western blot shown in Figure 5G and two additional independent western blots. Each line represents the change of Rps2 methylation at 30 °C vs. 16 °C within one individual blot. A pairwise t-test was performed to assess significances (** = p-value ≤ 0.01).
