# Supplementary material for: The N-terminal extension of ribosomal protein Rps2 shapes its early fate through Chaperone–Importin competition and arginine methylation
Source: J Biol Chem. 2026 Aug 25;302(10):113481. doi: 10.1016/j.jbc.2026.113481 (PMC13627120; doi:10.1016/j.jbc.2026.113481)
Supplement: Supplementary file 2 [file mmc2.pdf]

Supporting Information 2 – Supporting Tables

The N-terminal Extension of Ribosomal Protein Rps2 Shapes Its Early Fate through Chaperone–Importin Competition and Arginine Methylation

Maximilian Mack[1,2], Benjamin Pillet[3], Nicole Holton[4], Ingrid Zierler[1,2], Stephanie Himpich[4], Phyllis Jessen[1,2], Markus C. Wahl[4,5], Dieter Kressler[3], Brigitte Pertschy[1,2,*]

Table S1

Table S2

Table S1: List of yeast strains used in this study

Name	Genotype	Source
W303	*MAT***a***/MATα ade2-1/ade2-1, his3-11,15/his3-11,15, leu2-3,112/leu2-3,112, trp1-1/trp1-1, ura3-1/ura3-1, can1-100/can1-100*	(69)
Rps19a-TAP	[W303] *MAT***a**, *RPS19A*-TAP:: *HIS3*MX4	(10)
Rps19a-TAP *RPS2*-shuffle	[W303] *MAT***a**, *RPS19A*-TAP:: *HIS3*MX4, *rps2*Δ::kanMX4 [pRS316-*RPS2*]	This study
Rps19a-TAP *RPS2*-shuffle *hmt1*Δ	[W303] *MAT***a**, *RPS19A*-TAP:: *HIS3*MX4, *hmt1*Δ::hphNT1, *rps2*Δ::kanMX4 [pRS316-*RPS2*]	This study
Tsr4-TAP	[W303] *MAT***a**, *TSR4*-TAP:: *HIS3*MX4	(10)
Tsr1-TAP	[W303] *MAT***a**, *TSR1*-TAP:: *HIS3*MX4	This study
RPS2 shuffle	[W303] *MAT***a**, *rps2*Δ::kanMX4 [pRS316-*RPS2*]	This study
TSR4 shuffle	[W303] *MAT*α, *tsr4*Δ::*HIS3*MX4 [pRS316-*TSR4*]	(10)
RPS2 HMT1 double shuffle	[W303] *MAT***a**, *hmt1*Δ::hphNT1, *rps2* Δ::kanMX4 [pRS316-*RPS2*] [*pHT*Δ-HIS3-*CAN1-HMT1*]	This study
NOP58-yEmCherry	[W303] *MAT*α *NOP58*-yEmCherry::natNT2 *ade3*Δ::kanMX4	(7)
PJ69-4A	*MAT***a**, *trp1-901, leu2-3,112 ura3-52, his3-200, gal4*Δ, *gal80*Δ, *LYS2*::*GAL1-HIS3, GAL2-ADE2 met2::GAL7*-lacZ	(70)
PJ69-4A *hmt1*Δ	*MAT***a**, *trp1-901, leu2-3,112, ura3-52, his3-200, gal4*Δ, *gal80*Δ, *LYS2::GAL1-HIS3, GAL2-ADE2, met2::GAL7*-lacZ, *hmt1*Δ::*hphNT1*	This study

Table S2: List of plasmids used in this study

Name	Relevant information	Source
pCOLADuet-1	Kan^R, T7 promoter/lac operator	Novagen
pCOLADuet-1-*TSR4*-$(GA)_3$-His6	Kan^R, T7 promoter/lac operator; *TSR4*-His6 in MCS2	This study
pGEX-4TEV-*PSE1*	Amp^R, T7 promoter/lac operator; GST-*PSE1*	(72)
pRS314-*RPS2*	CEN, *TRP1*, $_P$*RPS2*, $_{ORF}$*RPS2*, $_T$*RPS2*	(10)
pRS314-*rps2*-R11K	CEN, *TRP1*, $_P$*RPS2*, $_{ORF}$*RPS2*, $_T$*RPS2*, R11K exchange	This study
pRS314-*rps2-8*	CEN, *TRP1*, $_P$*RPS2*, $_{ORF}$*rps2-8*, $_T$*RPS2*, G48D W144R K251E exchanges	This study
pRS316-*RPS2*	CEN, *URA3*, $_P$*RPS2*, $_{ORF}$*RPS2*, $_T$*RPS2*	(10)
pHTΔ-HIS3-CAN1-HMT1	CENΔ, *HIS3*, *CAN1*, $_P$*HMT1*, $_{ORF}$*HMT1*, $_T$*HMT1*	This study
pADH111-*RPS2*(1-145)-$(GA)_5$-3xyEGFP	CEN, *LEU2*, $_P$*ADH1*, $_{ORF}$*RPS2*(1-145) $_T$*ADH1*, C-terminal $(GA)_5$-3xyEGFP	(37)
pADH111-*rps2*(1-145)-RRK>A-$(GA)_5$-3xyEGFP	CEN, *LEU2*, $_P$*ADH1*, $_{ORF}$*RPS2*(1-145), $_T$*ADH1*, C-terminal $(GA)_5$-3xyEGFP, R95R97K99>A exchanges	(37)
pADH111-*rps2*(1-145)-R11A-$(GA)_5$-3xyEGFP	CEN, *LEU2*, $_P$*ADH1*, $_{ORF}$*RPS2*(1-145), $_T$*ADH1*, C-terminal $(GA)_5$-3xyEGFP, R11A exchange	This study
pADH111-*rps2*(1-145)-R11A-RRK>A-$(GA)_5$-3xyEGFP	CEN, *LEU2*, $_P$*ADH1*, $_{ORF}$*RPS2*(1-145), $_T$*ADH1*, C-terminal $(GA)_5$-3xyEGFP, R11A, R95R97K99>A exchanges	This study
pADH111-*rps2*(1-145)-R17A-$(GA)_5$-3xyEGFP	CEN, *LEU2*, $_P$*ADH1*, $_{ORF}$*RPS2*(1-145), $_T$*ADH1*, C-terminal $(GA)_5$-3xyEGFP, R17A exchange	This study
pADH111-*rps2*(1-145)-R17A-RRK>A-$(GA)_5$-3xyEGFP	CEN, *LEU2*, $_P$*ADH1*, $_{ORF}$*RPS2*(1-145), $_T$*ADH1*, C-terminal $(GA)_5$-3xyEGFP, R11A, R17A, R95R97K99>A exchanges $R_{95}R_{97}K_{99}$>A exchanges	This study

pADH111-*rps2*(1-145)-R11AR17A-(GA)$_5$-3xyEGFP	CEN, *LEU2*, $_{P}$*ADH1*, $_{ORF}$*RPS2*(1-145), $_{T}$*ADH1*, C-terminal (GA)$_5$-3xyEGFP, R11A, R17A exchanges	This study
pADH111-*rps2*(1-145)-R11A-R17A-RRK>A-(GA)$_5$-3xyEGFP	CEN, *LEU2*, $_{P}$*ADH1*, $_{ORF}$*RPS2*(1-145), $_{T}$*ADH1*, C-terminal (GA)$_5$-3xyEGFP, R11A R17A, R95R97K99>A exchanges	This study
pG4BDN22-*RPS2*(1-145)	CEN, *TRP1*, $_{P}$*ADH1*, $_{ORF}$*RPS2*(1-145), $_{T}$*ADH1*, N-terminal (GA)$_5$-G4BD	This study
pG4BDN22-*rps2*(1-145)-RRK>A	CEN, *TRP1*, $_{P}$*ADH1*, $_{ORF}$*RPS2*(1-145), $_{T}$*ADH1*, N-terminal (GA)$_5$-G4BD, R95R97K99>A exchanges	This study
pG4BDN22-*rps2*(1-145)-R11A-R17A	CEN, *TRP1*, $_{P}$*ADH1*, $_{ORF}$*RPS2*(1-145), $_{T}$*ADH1*, N-terminal (GA)$_5$-G4BD, R11K, R17K exchanges	This study
pG4BDN22-*rps2*(1-145)- R11A R17A-RRK>A	CEN, *TRP1*, $_{P}$*ADH1*, $_{ORF}$*RPS2*(1-145), $_{T}$*ADH1*, N-terminal (GA)$_5$-G4BD, R11A, R17A, R95R97K99>A exchanges	This study
pG4BDN112-*RPS2*	2µ, *TRP1*, $_{P}$*ADH1*, $_{ORF}$*RPS2*, $_{T}$*ADH1*, N-terminal (GA)$_5$-G4BD	(10)
pG4BDN112-*RPS2*(1-145)	2µ, *TRP1*, $_{P}$*ADH1*, $_{ORF}$*RPS2*(1-145), $_{T}$*ADH1*, N-terminal (GA)$_5$-G4BD,	(10)
pRS315-*TSR4*	CEN, *LEU2*, $_{P}$*TSR4*, $_{ORF}$*TSR4,* $_{T}$*TSR4*	(10)
pRS315-*tsr4-6*	CEN, *LEU2*, $_{P}$*TSR4*, $_{ORF}$*tsr4-6,* $_{T}$*TSR4,* L97P, N126D, K146E, T270A exchanges	This study
pG4ADC111-*TSR4*	CEN, *LEU2*, $_{P}$*ADH1*, $_{ORF}$*TSR4*, $_{T}$*ADH1*, C-terminal G4AD-HA	(10)
pG4ADC111-*tsr4-6*	CEN, *LEU2*, $_{P}$*ADH1*, $_{ORF}$*tsr4-6* $_{T}$*ADH1*, C-terminal G4AD-HA, L97P, N126D, K146E, T270A exchanges	This study
pG4BDC22-*RPS2*	CEN, *TRP1*, $_{P}$*ADH1*, $_{ORF}$*RPS2*, $_{T}$*ADH1*, C-terminal G4BD-c-Myc	(10)
pGAG4ADC181-*PSE1.302C* (*PSE1*(302-1089))	2µ, *LEU2*, $_{P}$*ADH1*, $_{ORF}$*PSE1*(302-1089), $_{T}$*ADH1*, C-terminal (GA)$_5$-G4AD	(37)

YEplac195-*PSE1*	2µ, *URA3*, $_{P}$*PSE1*, $_{ORF}$*PSE1*, $_{T}$*PSE1*	This study
pADH195-*TSR4*	2µ, *URA3*, $_{P}$*ADH1*, $_{ORF}$*TSR4*, $_{T}$*ADH1*	This study
pRS315-*HMT1*	CEN, *LEU2*, $_{P}$*HMT1*, $_{ORF}$*HMT1*, $_{T}$*HMT1*	This study
pRS315-*hmt1-G68R*	CEN, *LEU2*, $_{P}$*HMT1*, $_{ORF}$*HMT1*, $_{T}$*HMT1,* G68R exchange	This study
pRS314-*rps2*-G48D	CEN, *TRP1*, $_{P}$*RPS2*, $_{ORF}$*RPS2,* $_{T}$*RPS2,* G48D exchange	This study
pRS314-*rps2*-W144R	CEN, *TRP1*, $_{P}$*RPS2*, $_{ORF}$*RPS2,* $_{T}$*RPS2,* W144R exchange	This study
pRS314-*rps2*-K251E	CEN, *TRP1*, $_{P}$*RPS2*, $_{ORF}$*RPS2,* $_{T}$*RPS2,* K251E exchange	This study
pRS314-*rps2*-Y143C	CEN, *TRP1*, $_{P}$*RPS2*, $_{ORF}$*RPS2,* $_{T}$*RPS2,* Y143C exchange	This study

(P, T and ORF denote promoter, terminator, and open reading frame, respectively. Numbers indicated in parentheses after the ORF name refer to the amino acids encoded by the respective genes.)
